# The association between team job crafting and work engagement among nurses: a prospective cohort study

**DOI:** 10.1186/s40359-024-01538-7

**Published:** 2024-02-09

**Authors:** Mako Iida, Asuka Sakuraya, Kazuhiro Watanabe, Kotaro Imamura, Utako Sawada, Hiroto Akiyama, Yu Komase, Yuki Miyamoto, Norito Kawakami

**Affiliations:** 1 Department of Mental Health, Graduate School of Medicine, The University of Tokyo, 7-3-1, Hongo, Bunkyo-Ku, Tokyo, 113-0033 Japan; 2 Department of Psychiatric Nursing, Graduate School of Medicine, The University of Tokyo, 7-3-1, Hongo, Bunkyo-Ku, Tokyo, 113-0033 Japan; 3 Department of Digital Mental Health, Graduate School of Medicine, The University of Tokyo, 7-3-1, Hongo, Bunkyo-Ku, Tokyo, 113-0033 Japan; 4 Department of Public Health, Kitasato University School of Medicine, 1-15-1 Kitazato, Minami-ku, Sagamihara, Kanagawa, 252-0374 Japan; 5 Accenture Japan Ltd, Sumitomo Fudosan Azabu Juban Bldg., 1-4-1 Mita, Minato-ku, Tokyo, 108-0073 Japan; 6 Fujitsu Japan Limited, 1-5-2, Higashishinbashi, Minato-Ku, Tokyo, 105-7123 Japan

**Keywords:** Team job crafting, Collaborative job crafting, Work engagement

## Abstract

**Background:**

Team-level job crafting has been put forward as a method to promote nurses' mental health. However, a longitudinal association is unclear. Therefore, the objective of this study was to investigate the association between team job crafting at baseline and work engagement, work performance, psychological distress, and intention to leave at three-month and six-month follow-ups among Japanese hospital nurses. Also, whether an increase in the team job crafting during 3 or 6 months was associated with an increase in the work engagement during 3 or 6 months of individual nurses was examined.

**Methods:**

A multilevel prospective cohort study was conducted. Data were collected from nurses of five hospitals in Japan at baseline (T1) and follow-ups at 3-months (T2) and 6-months (T3). A total of 2,478 nurses were included. The team job crafting scale for nurses and its three subscales were measured for the independent variables. Ward-means were used as ward-level variables. The dependent variables were work engagement, work performance, psychological distress, and intention to leave. Hierarchical Linear Modeling (HLM) was used to examine the multilevel association. The study protocol was registered at the UMIN Clinical Trials Registry (ID = UMIN000047810) (May 22, 2022).

**Results:**

A total of 460 nurses completed the T1 survey (response rate = 18.6%), and data from 391 nurses nested in 30 wards were included in the analyses. The intraclass correlation coefficients (ICCs) at T1 were 0.02 for work engagement and 0.07 for team job crafting. The HLM revealed that ward-level team job crafting at T1 was not significantly associated with work engagement, work performance, psychological distress, and intention to leave at T2 or T3. The ward-level change (T3-T1) of “crafting for the task considering the team’s growth” (subscale for team job crafting) was significantly and positively associated with the change (T3-T1) in work engagement.

**Conclusions:**

Ward-level team job crafting at baseline did not predict nurses' work engagement, work performance, psychological distress, or intention to leave at a three-month or six-month follow-up. The impact of ward-level team job crafting may attenuate over several months.

**Supplementary Information:**

The online version contains supplementary material available at 10.1186/s40359-024-01538-7.

## Background

Throughout the world, nurses have an important role in hospital care and are known to engage in stressful work. Poor working conditions for nurses, such as a high workload, professional conflicts, the emotional cost of caring, and shift work, have a negative impact on mental health [1]. A recent study reported that one-third of nurses had depression and anxiety, and that half suffered from high emotional exhaustion and post-traumatic stress disorder [2]. The prevalence of mental health problems was reported to be higher than those of other occupations [3]. Improving a psychosocial work environment and mental health is necessary for nurses.

Recently, positive mental health has been a focus in the field of occupational health psychology [4]. Work engagement, one of the most well-known positive mental health indicators, is defined as a positive, fulfilling, work-related state of mind characterized by vigor, dedication, and absorption [5]. Work engagement consists of three dimensions: vigor (high levels of mental resilience and energy while working, as well as a willingness to invest effort in one's work and perseverance in the face of difficulties); dedication (a sense of significance, enthusiasm, inspiration, pride, and challenge); and absorption (being fully concentrated and deeply engrossed in one's work, whereby time passes quickly and makes it difficult to detach oneself from work [4, 5]. Work engagement refers to a more persistent and pervasive affective-cognitive state that is not focused on any specific object, event, individual, or behavior, rather than a momentary and specific state [5]. Previous studies have reported that work engagement was negatively related to symptoms of depression and anxiety [6], burnout [7], and psychological distress [8], and was positively related to job satisfaction and compassion satisfaction [7]. In the nursing setting, work engagement is valuable not only to individual nurses but also to patients and hospitals [9, 10]. For patient and hospital outcomes, work engagement was negatively associated with turnover intention from the hospital [7, 11] and positively associated with patient safety [12], work effectiveness [13], perceived care quality [14], and organizational commitment [15]. Therefore, work engagement plays a significant role in improving nurses’ health and job-related outcomes and patient outcomes.

According to the Job Demands-Resources (JD-R) model, job resources are proposed as an important predictor of work engagement [16]. Job resources were classified into three levels: task-level, interpersonal-level, and organizational-level [16]. In nursing settings, interpersonal-level and organizational-level job resources are more emphasized to improve work engagement among nurses [9]. Accordingly, improving such job resources could be effective.

Employees can improve their job resources by *job crafting* [17, 18]. Job crafting was conceptualized in 2001 by Wrzesniewski and Dutton. It is defined as the physical and cognitive changes individuals make in the tasks or relational boundaries of their work [17]. Job crafting involves three actions: task crafting, cognitive crafting, and relational crafting [17]. Job crafting was framed in the JD-R model in 2010 by Tims and Bakker [19], who defined job crafting as the changes employees make to balance their job resources (i.e., increasing structural and social job resources) and job demands (i.e., increasing challenging demands and decreasing hindering demands) with their abilities and needs [19, 20]. A previous cross-sectional study found a relationship between job crafting and work engagement among nurses [21]. The study also found that increasing the structural job resources aspects of individual job crafting showed a moderate association with work engagement, whereas increasing the social job resources aspects showed a weak association [21].

Recently, team-level job crafting, a new concept of job crafting, has been offered to increase nurses’ job resources [22]. In many situations, job crafting occurs at the team level as well as at the individual level because workers in a workgroup typically experience common events, engage in the same work processes, and interact with each other [23]. Leana et al. (2009) proposed "collaborative job crafting," which is a process of joint determination by a group of employees on how to alter their work to meet their shared objectives [23]. Tims et al. (2013) also proposed team job crafting with the definition of "the extent to which team members combine efforts to increase structural and social job resources as well as challenging job demands and to decrease their hindering job demands," according to the JD-R model [24]. Three aspects of team job crafting are reported among nurses [22] and were considered in line with the theory of Wrzesniewski & Dutton [17]. The first is "crafting for the task considering the team's growth," which is a similar concept to job crafting of increasing job resources and crafting task boundaries. Focusing on team growth may be a key part of the nurses' task-level team job crafting. The second aspect is "crafting for members' respect and reflection of meaningful work," which expands the individual job crafting concept of changing cognitive boundaries for unique features in team job crafting with interactions such as mutual respect. The third aspect is "crafting for smooth information sharing," which is similar to individual job crafting, focusing on changing relational boundaries. This aspect emphasizes information sharing among nurses because hospital ward nurses may need to share patient status, assessments, and plans. These three aspects reflect nurses' team job crafting separately from individual job crafting [22]. For nurses who work in a team, mutual support for workload management, better delegation practices, effective communication, enhanced interpersonal relationships, and better team orientation are essential to achieve good teamwork and high-quality care [25]. In this circumstance, team job crafting is expected to improve these job resources more efficiently and thus improve work engagement. While individual workers perform job crafting to fulfill their individual needs, team job crafting is accomplished by team members to achieve team goals or objectives [17, 22, 26]. Thus, team job crafting is expected to improve interpersonal and organizational-level job resources.

A cross-sectional association between team job crafting and work engagement has been reported among general (non-healthcare) workers. For example, team job crafting was reported to be associated with individual work engagement, job satisfaction, work performance, organizational commitment, and quality of care [23, 24, 27, 28]. For nurses, there were two previous studies. A cross-sectional study found a positive association between team job crafting and individual-level work engagement among medical professionals from a Chinese public hospital [29]. Another study reported that baseline team job crafting was related to team-level work engagement at one-month follow-up among clinicians, including physicians and nurses in Vietnam [30]. Therefore, team job crafting may also be associated with better work engagement among nurses.

However, previous studies investigated only cross-sectional or short-term (one-month) associations. The evidence of cross-sectional studies has the possibility of causal reversal between its association. While there was only one longitudinal study that clarified the association between team job crafting and work engagement, the dependent variable was team-level work engagement. Thus, the longitudinal relationship between ward-level team job crafting and individual-level work engagement has not been examined. Also, more than the one-month longitudinal relationship between team job crafting and work engagement is unknown. Investigating the three-month or six-month association may yield additional evidence to support long-term effects of team job crafting behavior in a ward on work engagement and work performance of nurses in a ward. These findings would help nursing managers recognize the importance of team job crafting as an organizational-level measure intended to improve mental health.

The association between team job crafting and work engagement may vary depending on the organizational climate. One aspect of the organizational climate is workplace social capital, which is described as the contextual psychosocial elements of a workplace characterized by interpersonal trust and reciprocity rules [31]. Psychological safety could be another aspect of organizational climate, defined as the shared belief by team members that the team can safely take risks [32]. It is argued that job crafting behavior improves positive affective well-being when the response from colleagues is supportive. Conversely, it improves negative affective well-being when the reaction from colleagues is hostile [33]. Therefore, team job crafting behavior would be more readily accepted by team members in a workplace with a trustworthy and reciprocal or safe organizational climate [32, 34]. On the other hand, team job crafting would be less acceptable in a ward with an unaccommodating organizational climate because members may feel forced to do it, making it challenging to improve work engagement. Thus, team job crafting is more likely associated with work engagement in a ward with greater workplace social capital and psychological safety.

Additionally, team job crafting may be positively associated with work performance and negatively associated with psychological distress and intention to leave. Work performance could be measured by self-report by asking a respondent to rate his/her work performance compared to that of most coworkers [35, 36]. Previous studies reported significant positive associations between team job crafting and work performance [23, 24, 28, 30]. Psychological distress refers to non-specific emotional distress, such as depression and anxiety [37], and intention to leave refers to a perception of leaving the organization and could be a strong predictor of turnover [38]. Previous studies reported significant negative associations between individual job crafting and psychological distress and intention to leave [39, 40]. However, the longitudinal association between team job crafting and these outcomes was unknown.

This study aimed to investigate the longitudinal association between ward-level team job crafting at baseline and individual-level work engagement at three-month and six-month follow-ups among Japanese nurses. Also, the subgroup analyses investigated the difference in the association between ward-level team job crafting at baseline and individual-level work engagement at three-month and six-month follow-ups by organizational climate (i.e., high/low workplace social capital wards and high/low psychological safety wards). The associations between team job crafting at baseline and individual-level work performance, psychological distress, and intention to leave at three-month and six-month follow-ups were also examined as secondary outcomes.

In addition to examining the association between baseline team job crafting and work engagement in the follow-up surveys, the change-to-change relations among ward-level team job crafting and individual-level work engagement from the baseline to the follow-up survey by using scores of differences would also be helpful. A previous researcher demonstrated that investigating hypotheses in which changes in one construct are are related to changes in another construct is a good way to find connections between dynamic processes [41]. Therefore, We investigated whether an increase in the ward-level team job crafting score has an effect with an increase in the work engagement scores of individual nurses nested in the ward.

### Hypothesis

- H1: Ward-level team job crafting (total score and each of three subscales) at baseline would be positively associated with individual-level work engagement at the three-month and six-month follow-ups.
- H2: The degree of association between ward-level team job crafting (total score and each of three subscales) at baseline and individual-level work engagement at the three-month and six-month follow-ups would be greater in wards with higher degrees of workplace social capital and psychological safety at baseline.
- H3: Ward-level team job crafting (total score and each of three subscales) at baseline would be positively associated with individual-level work performance and negatively associated with psychological distress and intention to leave at the three-month and six-month follow-ups.
- H4: Ward-level change of team job crafting (total score and each of three subscales) from baseline to three-month and six-month follow-ups would be positively associated with an individual-level change of work engagement.

## Methods

### Study design

This was a prospective cohort study. Multilevel data were collected three times from hospital wards and nurses and midwives working at target hospitals. The first survey was conducted from December 2021-January 2022 (T1), the second survey was conducted in March 2022 (T2), and the third survey was conducted from June-July 2022 (T3). A self-report questionnaire assessed individual-level variables. The study protocol was registered at the UMIN Clinical Trials Registry (ID = UMIN000047810). The study was approved by The Research Ethics Committee of the Graduate School of Medicine/Faculty of Medicine at the University of Tokyo [No. 2018175NI-(3)]. This study has been reported according to the Strengthening the Reporting of Observational Studies in Epidemiology [42] (see Additional file 1).

### Participants

Japanese hospitals having inpatient wards were approached using snowball sampling methods. We approached medium to large-scale hospitals, chosen through the connections of the first author (MI). The first author sent an e-mail or letter with an explanation of the study and an invitation to participate and negotiated with the nursing department director. After the nursing department director agreed to participate in the study, surveys were given through a web-based questionnaire or a paper questionnaire, according to the policy of the director. The inclusion criteria of the wards were (a) wards in Japanese hospitals and (b) employed two or more nurses. Outpatient wards and clinics were excluded. The inclusion criteria of individuals were nurses and midwives working in the included hospital wards. Nurses and midwives working in outpatient wards and clinics were excluded. Informed consent was obtained by all participants using the consent form in the paper or online questionnaire.

### Variables

#### Independent variables

Team job crafting was measured by the Japanese Team Job Crafting Scale [22]. Internal consistency reliability and concept validity were determined to be acceptable. The scale includes 13 items (e.g., "The proficiency levels of newcomers are properly understood to help them grow", “positive events are shared among team members in order to reaffirm the value of our work,” and “a patient’s information is shared among team members even outside of meetings in order to provide better care for the patient as a team.”) measured on a five-point response scale (1 = not at all, 5 = extremely). Higher scores indicate higher team job crafting behavior in a ward. The total score, as well as each subscale score, was calculated by dividing the sum of item scores by the number of the items. The mean score of the three subscales (overall) was calculated as individual-level scores. The mean score of each of the three subscales (subscale 1, subscale 2, subscale 3) was also calculated. Ward-level scores were calculated by the ward-mean score (overall, subscale 1, subscale 2, subscale 3). Both individual- and ward-level scores were grand-mean centered on establishing a useful zero point [43].

#### Dependent variables

##### Work engagement

As the primary dependent variable, work engagement was assessed using the 9-item Japanese version of the Utrecht Work Engagement Scale (UWES) [44, 45]. The UWES has nine items (e.g., "At my job, I feel strong and vigorous"). Every item was measured on a seven-point Likert scale ranging from 0 (Never) to 6 (Always), with higher scores meaning higher work engagement. Previously, the reliability and validity of the Japanese UWES were examined [45]. Mean scores for each of the nine items were calculated.

##### Work performance

The Japanese short version of the WHO Health and Work Performance Questionnaire (WHO-HPQ) was used to evaluate work performance [36]. The reliability and validity of this self-reporting questionnaire were evaluated and found to be consistent with another self-reported presenteeism scales [36]. The scale consists of one item that rates an individual's total job performance over the preceding month ranging from 0 (Worst) to 10 (Best). Higher scores mean higher work performance.

##### Psychological distress

Psychological distress was assessed with the Japanese version of the K6 questionnaire, which consists of six items and asks respondents how frequently they had experienced psychological distress symptoms in the previous 30 days [46]. The reliability and validity were evaluated [46]. On a five-point Likert scale, responses range from 0 (None of the time) to 4 (All of the time), with higher scores indicating higher psychological distress. A total score (0 to 24) was calculated.

##### Intention to leave

Intention to leave was measured with the four-items of the Japanese version of the intention to leave scale (e.g., "If it would have been easier to change employers, I would have quit a long time ago") [38, 47]. The participants responded to each item on a five-point Likert scale ranging from 1 (I disagree completely) to 5 (I agree completely). Higher scores indicate higher intention to leave. The mean score of the items was calculated.

##### Confounding variables

Job stressors, workplace social support, and demographic variables were measured as confounding variables. For the multilevel analysis, these variables were treated as individual-level and grand-mean-centered.

##### Job demands, job control, supervisor/coworker support

Job demands, job control, and supervisor/coworker support were measured by items from the Brief Job Stress Questionnaire, which is widely used to assess psychosocial factors at work in Japan [48, 49]. All items were scored on a four-point Likert scale ranging from 1 (Not at all) to 4 (Definitely), with higher scores indicating higher job demands, job control, and supervisor/coworker support. The scores of the four scales (i.e., job demands, job control, supervisor support, and coworker support) were calculated as a sum of item scores of each scale divided by the number of items.

Test–retest reliability and validity were reported to be at adequate levels [49].

##### Effort-Reward imbalance

Effort-reward imbalance was measured by the validated Japanese short version of the effort-reward imbalance (ERI) questionnaire [50, 51]. The effort scale consists of three items related to work pressure and immersion, and the reward scale consists of seven items related to financial, esteem-related, and organizational rewards. The items were rated on a 4-point Likert scale (1 = strongly disagree, 4 = strongly agree). Higher scores mean higher effort and reward. Sum scores for effort and reward were calculated, and higher scores indicate more effort and reward, respectively. A high ERI ratio reflects a lack of reciprocity between efforts and rewards (high cost/low gain).

##### Demographic variables

Gender (men, women, other, no response), age (20–29, 30–39, 40–49, 50–59, 60-), educational status (professional school, university/graduate school, other), marital status (not married, married, no response), and professional experience (years) (1–4, 5–9, 10–19, 20–29, 30-) were measured. Adjusting for confounding variables in the analyses, "no response" selected for the questions about gender and marital status was included in the "no response" category.

#### Variables for the subgroup analyses

##### Workplace social capital

Workplace social capital was measured by the Japanese version of the Workplace Social Capital Scale [52, 53]. The Japanese version was preliminarily tested and proved to have an acceptable level of internal consistency reliability and construct validity [53]. The scale includes eight items (e.g., "We have a 'we are together' attitude") measured on a five-point Likert scale, and the mean score was calculated. Higher scores indicate higher workplace social capital. The ward-level score was calculated by the ward mean of the individual score and grand-mean centered.

##### Psychological safety

Psychological safety was measured by the Japanese version of the Psychological safety scale [32, 54]. Internal consistency reliability and validity were reported at an acceptable level. The scale consists of seven items (e.g., “Members of this team are able to bring up problems and tough issues”) measured on a seven-point Likert scale, and the mean score was calculated. Higher scores mean higher psychological safety. The ward-level score was calculated by the ward mean of the individual score and grand-mean centered.

### Sample size calculation

The target sample size was calculated by accounting for intraclass correlations (ICC) of the organizational outcomes [55]. Sample sizes should be multiplied by the design effect (1 + [m − 1] ρ), where m is the average cluster size, and ρ is ICC. In a previous study, ICC for work engagement among Japanese nurses was 0.09, and the number of clusters was set to 22 [56]. The effect size of team job crafting was estimated at 0.20 based on a validation study of the team job crafting scale [22]. Based on these estimations, 2277 participants from 103 wards were needed at an alpha error rate of 0.05 and a beta error rate of 0.20 using G*Power version 3.1.9.6 [57].

### Statistical analysis

In the analyses, wards with one or fewer respondents were additionally excluded because ward-level team job crafting, calculated as an average of ward members' ratings, might not be stable and it is more likely to be affected by individual characteristics of the ward members [58].

First, descriptive statistics for the variables and the ICCs for team job crafting, work engagement, performance, psychological distress, intention to leave, workplace social capital, and psychological safety were calculated. The design effect was also calculated to assess a need for multilevel framework data analysis. Then, HLM was conducted to investigate multilevel relationships between team job crafting at T1 and dependent variables at T2 and T3 to test the first, second, and third hypotheses.

Multilevel regression modeling techniques [59, 60] were employed to investigate the hypotheses. The data were conceptualized as a two-level model, consisting of an individual at the first level and a ward at the second level. Four steps of multilevel analyses at T2 and T3 were conducted. The first step of the analysis was to determine the variance at individual and ward levels without explanatory variables (null model: Model 0). This model contains random groups and random variation within groups. Then, individual-level and ward-level team job crafting were entered into the null model (crude model: Model 1), the T1 dependent variable, demographic variables, and effort-reward imbalance were entered into Model 1 (T1 dependent variable, demographic variables, and effort-reward imbalance adjusted model: Model 2), and variables of job demands and job resources (job control and supervisor/coworker support) were entered into Model 2 (job demand and resource variables adjusted model: Model 3). The overall team job crafting score was included as independent variables for Model 1, Model 2, and Model 3. Akaike information criterion (AIC) was calculated to check the best-fit model. The scores of three team job crafting subscales were included only in the best-fitted model. The equation for the adjusted model (Model 3) could be explained as follows.

- Level 1 (individual-level)
  $${Y\;(work\;engagement\;at\;T2\;and\;T3)}_{ij}= {\beta }_{0j} + {\beta }_{1j} * {(team\;job\;crafting)}_{ij} +{\beta }_{2j} * {(T1\;work\;engagement)}_{ij} + {\beta }_{3j} * {(gender)}_{ij} + {\beta }_{4j} * {(age)}_{ij}+ {\beta }_{5j} * {(education)}_{ij}+ {\beta }_{6j} * {(marital\;status)}_{ij}+ {\beta }_{7j} * {(job\;demand)}_{ij}+ {\beta }_{8j} * {(job\;control)}_{ij} + {\beta }_{9j} * {(supervisor\;support)}_{ij} + {\beta }_{0j} * {(coworker\;support)}_{ij }+ {\beta }_{11j }* {(effort-reward\;imbalance)}_{ij} + {e}_{ij}$$
- Level 2 (ward-level)
  $${\beta }_{0j} = {\gamma }_{00} + {\gamma }_{01}* {(team\;job\;crafting)}_{0j} + {u}_{0j}$$

Note: Y_ij_ means the score of work engagement of individual nurse i who attends wards j. e and u are assumed to be error terms.

To test the fourth hypothesis, HLM were also conducted. The ward-level change in team job crafting from T1 to T2 and from T1 to T3 was put into the model as independent variables. Each score of three team job crafting subscales as well as total score were included the analyses. The individual-level change in work engagement from T1 to T2 and from T1 to T3 was put into the model as dependent variables. Additionally, the association between ward-level change in team job crafting from T1 to T2 and individual-level change in work engagement from T2 to T3 was examined.

Nurses with no data on wards have been excluded from the analyses. Using the multiple imputation approach for parameter estimation [61, 62], the wards and nurses with partially missing values, except for the ward data, or who dropped out of the study during follow-up were included in the evaluated model. An independent variable, dependent variables, and confounding variables were used for the imputation. A total of 100 simulated completed datasets were generated by predictive mean matching. R version 4.2.1 with mice package for the multiple imputations was used for each analysis [63].

### Subgroup analyses

First subgroup analyses were conducted separately for wards with high scores (higher than or equal to the median) and low scores (less than the median) of workplace social capital and psychological safety at baseline. Although previous studies used a quartile point for cut-off for workplace social capital [64, 65] and psychological safety [66], this study used the median score because of the smaller sample size.

### Changes to protocol

Two changes were made to the protocol registered at the UMIN Clinical Trials Registry (UMIN-CTR) (ID = UMIN000047810). First, the original plan was only for analysis that investigates the association between baseline team job crafting and work engagement at three-month and six-month follow-ups. However, because the survey timing crossed the fiscal year and the pandemic of COVID-19 infection, a decrease in team job crafting was expected. Thus, an analysis of change-to-change relations of team job crafting and work engagement was added. Second, in the protocol, a quartile point is used for cut-off for workplace social capital and psychological safety. However, this study used the median score for the cut-off point because of the smaller sample size for wards.

## Results

### Participants

**Fig. 1:**
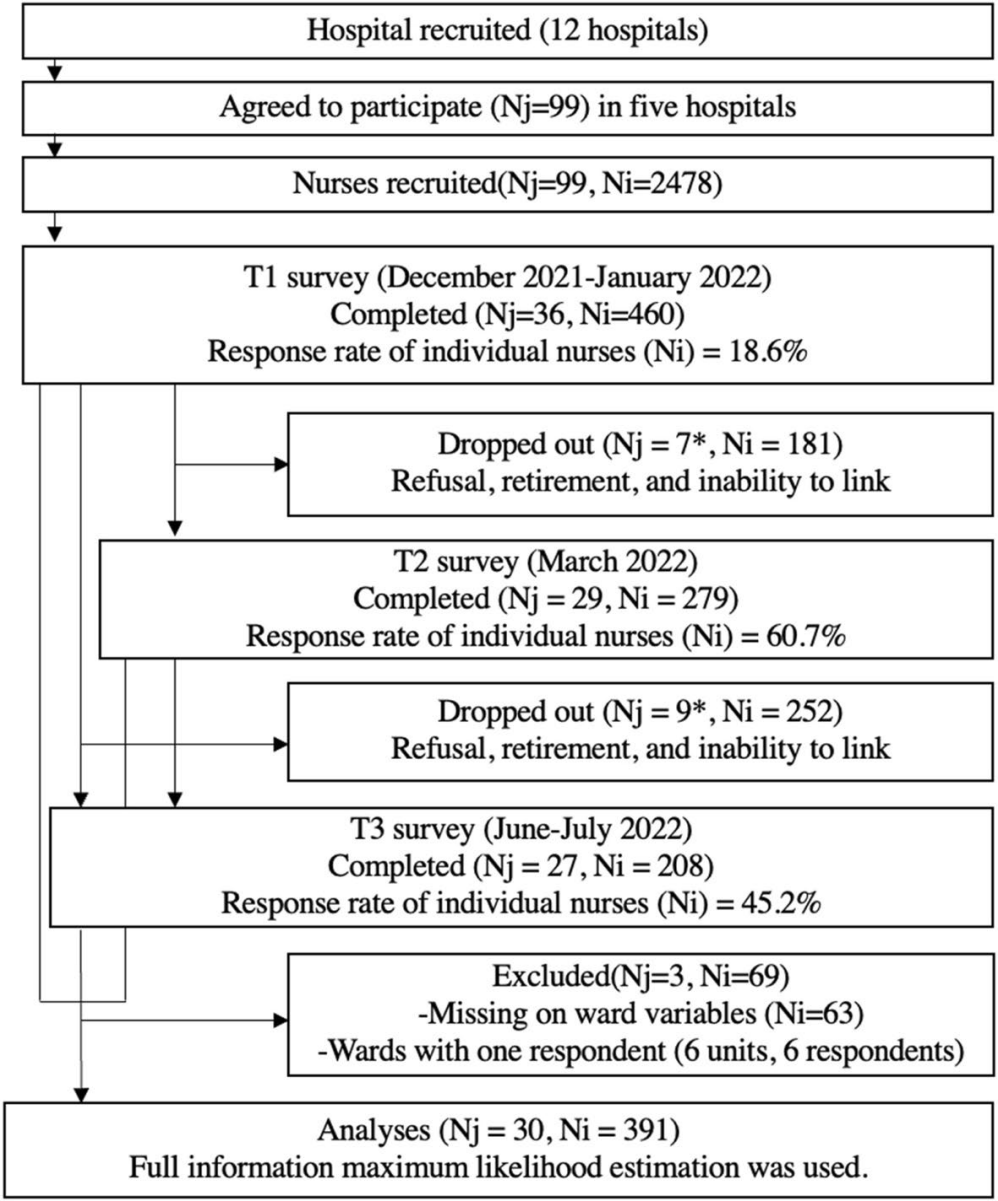
Participation flow chart for wards and nurses Note. Nj = the number of wards; Ni = the number of nurses. *Because of individual dropped outs, no nurses were included in the ward

Figure 1 shows the study's participation flow chart of the wards and nurses. A total of twelve hospitals were invited to participate in the study, and five hospitals agreed to participate. Of these, 2478 nurses nested in 99 wards were included. A total of 460 nurses nested in 36 wards completed the T1 survey (response rate of individual nurses = 18.6%). Of 460 nurses, 63 nurses were excluded due to missing ward variables. Also, six nurses in six wards with only one respondent were additionally excluded. The average numbers of nurses who completed the T1 survey from each ward were 24 (Min: two respondents and Max: 36 respondents in a ward). At the T2 survey, 29 wards representatives and 279 nurses completed the survey (response rate of individual nurses = 60.7%). Seven wards and 181 nurses refused or dropped out from T1 to T2. At the T3 survey, 27 wards representatives and 208 nurses completed the survey (response rate of individual nurses = 45.2%). Nine wards and 252 nurses refused or dropped out from T1 to T3. Nurses who responded to the T1 survey were included in the analyses even if they dropped out at the T2 or T3 survey. The actual collected and analyzed data was 391 participants from 30 wards, and the post hoc statistical power of analysis (1—β) would be 0.20.

### Participant characteristics

Table 1 shows the characteristics of the 30 wards and 391 nurses at T1. Among the wards, 14 (46.7%) included 2–9 respondents, 8 (26.7%) included 10–19 respondents, 7 (23.3%) included 20–29 respondents, and 1 (3.3%) included 30–39 respondents. Types of clinical departments of wards were internal medicine wards (4), surgical wards (4), psychiatric wards (13), pediatric wards (2), and mixed wards or other (7). Of the nurses, most (78.5%) were female.

**Table 1:** Baseline characteristics of wards and nurses at hospitals which participated in the study (Nj = 30, Ni = 391)

	N (%)	Mean (SD)	Min–Max
**Ward-level variables (Nj = 30)**			
Respondents in a ward			
2–9	14 (46.7)		
10–19	8 (26.7)		
20–29	7 (23.3)		
30–39	1 (3.3)		
Clinical department of wards			
Internal medicine wards	4 (13.3)		
Surgical wards	4 (13.3)		
Psychiatric wards	13 (43.3)		
Pediatric wards	2 (6.7)		
Mixed wards, other	7 (23.3)		
**Individual-level variables (Ni = 391)**			
Gender			
Men	82 (21.0)		
Women	307 (78.5)		
Other	1 (0.3)		
No response	0 (0.0)		
Missing	1 (0.3)		
Age		36.6 (11.8)	21–70
20–29	146 (37.3)		
30–39	89 (22.8)		
40–49	86 (22.0)		
50–59	49 (12.5)		
60-	16 (4.2)		
Missing	5 (1.3)		
Education			
Professional school	339 (86.7)		
University/Graduate school	49 (12.5)		
Other	2 (0.5)		
Missing	1 (0.3)		
Marital status			
Not married	205 (52.4)		
Married	180 (46.0)		
No response	3 (0.8)		
Missing	3 (0.8)		
Professional experience (years)			
1–4	88 (22.5)		
5–9	73 (18.7)		
10–19	95 (24.3)		
20–29	63 (16.1)		
30-	35 (9.0)		
Missing	37 (9.5)		

*Nj* The number of wards, *Ni* The number of individuals

Table 2 presents the means and SDs of the independent variables at baseline and dependent variables at baseline, 3-month, and 6-month follow-up. Table 3 shows the intraclass correlation coefficients (ICCs) for the study variables and individual-level and ward-level correlations among variables. ICCs were calculated to estimate the proportion of variance explained at each level of the studies [67]. The results showed that 95% of the variance in T2 work engagement and 99% in T3 work engagement is explained by variables from individuals, and 5% of the variance in T2 and 1% in T3 work engagement is explained from wards. The design effect was 1.60, which could not exceed 2.0. On the other hand, ICCs for independent (i.e., team job crafting and each domain of team job crafting) ranged from 0.03 to 0.12, indicating that 3–12% of the variances were explained by these ward-level variables. Additionally, ICCs for the change in team job crafting scores between T1 and T2 ranged from 0.01 to 0.04, and between T1 and T3 ranged from 0.01 to 0.03. The change in work engagement scores between T1 and T2 was 0.15, and between T1 and T3 was 0.05.

**Table 2:** Means, standard deviations of independent variables, variables for subgroup analyses and dependent variables (Nj = 30, Ni = 391)

		Baseline (T1)			3-month follow-up (T2)			6-month follow-up (T3)		
Individual-level variables	Range	Ni	Mean	SD	Ni	Mean	SD	Ni	Mean	SD
Team job crafting (total)	1–5	379	3.20	0.64	267	3.20	0.68	198	3.17	0.61
Crafting for task	1–5	382	3.34	0.66	268	3.28	0.70	200	3.29	0.59
Crafting for respect	1–5	389	2.96	0.77	270	3.04	1.00	200	2.96	0.75
Crafting for information share	1–5	387	3.31	0.72	271	3.31	0.69	198	3.26	0.69
Work engagement	0–6	384	2.48	1.00	268	2.53	1.01	201	2.42	1.13
Work performance	0–11	382	5.07	1.76	268	5.38	1.76	200	4.90	1.93
Psychological distress	0–24	388	5.30	4.89	269	5.13	4.98	202	5.55	5.07
Intention to leave	1–4	388	2.80	0.52	270	2.80	0.52	203	2.80	0.56
Workplace social capital	1–4	387	2.95	0.58	-	-	-	-	-	-
Psychological safety	1–7	385	4.44	0.88	-	-	-	-	-	-
Ward-level variables	Range	Nj	Mean	SD	Nj	Mean	SD	Nj	Mean	SD
Team job crafting (total)	1–5	30	3.20	0.24	26	3.20	0.24	25	3.13	0.26
Crafting for task	1–5	30	3.33	0.21	26	3.28	0.25	25	3.27	0.28
Crafting for respect	1–5	30	2.96	0.32	26	3.03	0.34	25	2.91	0.31
Crafting for information share	1–5	30	3.31	0.26	26	3.20	0.24	25	3.22	0.29
Workplace social capital	1–5	30	2.95	0.30	-	-	-	-	-	-
Psychological safety	1–7	30	4.44	0.37	-	-	-	-	-	-

*Nj* The number of wards, *Ni* The number of individuals

**Table 3:** Intra class correlations (ICCs) for each variable and multilevel correlations among ward-level variables (Nj = 30, Ni = 391)

	ICC [95%CI]	1	2	3	4	5	6	7	8	9	10	11	12	13	14	15	16	17	18
1. Team job crafting (T1)	0.07 [0.02–0.17]	1.00	0.87*	0.91*	0.90*	0.57*	0.43*	0.41*	0.32*	0.30*	0.12*	0.24*	0.23*	-0.30*	-0.26*	-0.28*	-0.37*	-0.29*	-0.32*
2. Crafting for task (T1)	0.03 [-0.01–0.12]	0.73*	1.00	0.68*	0.67*	0.46*	0.34*	0.35*	0.29*	0.28*	0.12*	0.22*	0.23*	-0.25*	-0.22*	-0.24*	-0.31*	-0.23*	-.028*
3. Crafting for respect (T1)	0.12 [0.05–0.23]	0.94*	0.56*	1.00	0.73*	0.51*	0.39*	0.41*	0.34*	0.29*	0.08	0.20*	0.22*	-0.25*	-0.22*	-0.21*	-0.36*	-0.30*	-.031*
4. Crafting for information (T1)	0.07 [0.01–0.17]	0.91*	0.48*	0.82*	1.00	0.55*	0.42*	0.37*	0.24*	0.23*	0.13*	0.22*	0.14*	-0.30*	-0.24*	-0.27*	-0.33*	-0.26*	-.026*
5. Workplace social capital (T1)	0.21 [0.12–0.37]	0.74*	0.54*	0.68*	0.69*	1.00	0.51*	0.40*	0.34*	0.37*	0.11*	0.11	0.17*	-0.29*	-0.22*	-0.28*	-0.42*	-0.31*	-0.35*
6. Psychological safety (T1)	0.12 [0.05–0.23]	0.74*	0.47*	0.66*	0.78*	0.79*	1.00	0.32*	0.25*	0.32*	0.25*	0.15*	0.34*	-0.39*	-0.39*	-0.33*	-0.40*	-0.33*	-0.37*
7. Work engagement (T1)	0.02 [-0.02–0.09]	0.25*	0.36*	0.24*	0.10	0.21*	0.14*	1.00	0.83*	0.79*	0.43*	0.44*	0.45*	-0.31*	-0.21*	-0.37*	-0.51*	-0.47*	-0.49*
8. Work engagement (T2)	0.05 [-0.01–0.15]	0.10	0.08	0.15*	0.04	0.16*	0.22*	0.62*	1.00	0.82*	0.39*	0.47*	0.47*	-0.27*	-0.23*	-0.41*	-0.44*	-0.53*	-0.50*
9. Work engagement (T3)	0.01 [-0.05–0.12]	0.29*	0.12*	0.34*	0.28*	0.30*	0.27*	0.64*	0.65*	1.00	0.39*	0.44*	0.59*	-0.36*	-0.33*	-0.47*	-0.44*	-0.52*	-0.52*
10. Work performance (T1)	0.003 [-0.03–0.07]	0.09	0.05	0.01	0.20*	0.12*	0.22*	0.36*	0.14*	0.29*	1.00	0.51*	0.54*	-0.37*	-0.25*	-0.36*	-0.14*	-0.19*	-0.19*
11. Work performance (T2)	0.04 [-0.02–0.14]	0.02	-0.01	0.09	-0.04	0.12*	0.04	0.34*	0.46*	0.42*	0.14*	1.00	0.51*	-0.43*	-0.34*	-0.45*	-0.21*	-0.21*	-0.24*
12. Work performance (T3)	0.01 [-0.05–0.12]	-0.01	-0.08	0.02	0.00	-0.04	0.01	0.49*	0.35*	0.63*	0.49*	0.39*	1.00	-0.38*	-0.34*	-0.43*	-0.27*	-0.31*	-0.26*
13. Psychological distress (T1)	0.03 [-0.01–0.10]	-0.16*	-0.09	-0.16*	-0.16*	-0.13*	-0.37*	-0.53*	-0.55*	-0.43*	-0.35*	-0.40*	-0.48*	1.00	0.73*	0.63*	0.34*	0.27*	0.27*
14. Psychological distress (T2)	0.06 [-0.002–0.18]	-0.11*	0.10	-0.17*	-0.17*	0.03	-0.25*	-0.38*	-0.45*	-0.49*	-0.29*	-0.42*	-0.44*	0.76*	1.00	0.67*	0.25*	0.25*	0.22*
15. Psychological distress (T3)	-0.05 [-0.08–0.03]	-0.27*	0.05	-0.32*	-0.35*	-0.10	-0.35*	-0.34*	-0.23*	-0.59*	-0.40*	-0.36*	-0.69*	0.56*	0.76*	1.00	0.34*	0.36*	0.45*
16. Intention to leave (T1)	0.14 [0.06–0.27]	-0.40*	-0.32*	-0.34*	-0.39*	-0.47*	-0.44*	-0.57*	-0.36*	-0.55*	-0.40*	-0.08	-0.16*	0.41*	0.28*	0.32*	1.00	0.64*	0.63*
17. Intention to leave (T2)	0.21 [0.10–0.38]	-0.39*	-0.14*	-0.40*	-0.43*	-0.53*	-0.57*	-0.36*	-0.50*	-0.50*	-0.37*	-0.05	-0.12*	0.35*	0.35*	0.34*	0.83*	1.00	0.61*
18. Intention to leave (T3)	0.20 [0.08–0.38]	-0.38*	-0.13*	-0.38*	-0.46*	-0.52*	-0.51*	-0.09	-0.19*	-0.42*	-0.34*	-0.22*	-0.16*	0.10*	0.30*	0.40*	0.54*	0.69*	1.00

Upper triangular matrix indicates individual-level correlations, and lower triangular matrix indicates ward-level matrix

*Nj* the number of wards, *Ni* the number of individuals. *ICC* intra-class correlation coefficient interval

^*^ *p* < 0.05

### Longitudinal association between team job crafting and work engagement

Tables 4 and 5 is the main results of HLM on work engagement at T2 and T3, respectively. Almost 95% of the variance was situated at the individual level, larger than the ward level (5%). The analyses revealed that ward-level team job crafting at T1 have no significant relationships on work engagement at T2 (*γ*_*01*_ = -0.22 [SE = 0.44], *p* = 0.615) and T3 (*γ*_*01*_ = -0.13 [SE = 0.58], *p* = 0.817). No significant association was found between any subscale of ward-level team job crafting at T1 and work engagement at T2 and T3.

**Table 4:** Multilevel association between individual- and ward-level team job crafting and work engagement at T2 (Nj = 30, Ni = 391)

**Fixed effects**	Model 0 (Null model)		Model 1 (Crude model)		Model 2 (adjusted^a^)		Model 3 (adjusted^b^)		Model 3a (adjusted^a^)		Model 3b (adjusted^a^)		Model 3c (adjusted^a^)	
	Coefficient (SE)	*p* value	Coefficient (SE)	*p* value	Coefficient (SE)	*p* value	Coefficient (SE)	*p* value	Coefficient (SE)	*p* value	Coefficient (SE)	*p* value	Coefficient (SE)	*p* value
Intercept	2.62 (0.66)	< 0.001	2.62 (0.66)	< 0.001	2.64 (0.65)	< 0.001	2.63 (0.65)	< 0.001	2.64 (0.65)	< 0.001	2.64 (0.65)	< 0.001	2.64 (0.66)	< 0.001
Individual-level	Coefficient (SE)	*p* value	Coefficient (SE)	*p* value	Coefficient (SE)	*p* value	Coefficient (SE)	*p* value	Coefficient (SE)	*p* value	Coefficient (SE)	*p* value	Coefficient (SE)	*p* value
Team job crafting			0.35 (0.15)	0.021	-0.05 (0.15)	0.720	-0.10 (0.15)	0.515						
Crafting for task									-0.02 (0.16)	0.882				
Crafting for respect											0.004 (0.11)	0.965		
Crafting for information													-0.09 (0.12)	0.447
Ward-level	Coefficient (SE)	*p* value	Coefficient (SE)	*p* value	Coefficient (SE)	*p* value	Coefficient (SE)	*p* value	Coefficient (SE)	*p* value	Coefficient (SE)	*p* value	Coefficient (SE)	*p* value
Team job crafting			-0.26 (0.47)	0.587	-0.19 (0.45)	0.678	-0.22 (0.44)	0.615						
Crafting for task									-0.32 (0.45)	0.475				
Crafting for respect											-0.13 (0.36)	0.722		
Crafting for information													-0.08 (0.47)	0.859
**Random effects**	Coefficient		Coefficient		Coefficient		Coefficient		Coefficient		Coefficient		Coefficient	
Intercept	0.09		0.08		0.11		0.10		0.12		0.11		0.11	
Residual variance	0.90		0.91		0.90		0.89		0.88		0.88		0.88	
AIC	1380.254		1328.841		1292.572		1300.459		Coefficient (SE)	*p* value	Coefficient (SE)	*p* value	Coefficient (SE)	*p* value

*Nj* the number of wards, *Ni*, the number of individuals

^a^Adjusted by age, gender, educational status, marital status, nurse experiences, effort-reward imbalance, T1 work engagement

^b^Adjusted by T1 work engagement

^b^Adjusted by age, gender, educational status, marital status, nurse experiences, effort-reward imbalance, T1 work engagement, job demand, job control, supervisor support, coworker support

**Table 5:** Multilevel association between individual- and ward-level team job crafting and work engagement at T3. (Nj = 30, Ni = 391)

	Model 0 (Null model)		Model 1 (Crude model)		Model 2 (adjusted^a^)		Model 3 (adjusted^b^)		Model 3a (adjusted^b^)		Model 3b (adjusted^b^)		Model 3c (adjusted^b^)	
**Fixed effects**	Coefficient (SE)	*p* value	Coefficient (SE)	*p* value	Coefficient (SE)	*p* value	Coefficient (SE)	*p* value	Coefficient (SE)	*p* value	Coefficient (SE)	*p* value	Coefficient (SE)	*p* value
Intercept	2.56 (0.99)	< 0.001	2.55 (0.99)	< 0.001	2.56 (0.36)	< 0.001	2.56 (0.36)	< 0.001	2.56 (0.98)	< 0.001	2.56 (0.10)	< 0.001	2.57 (0.99)	< 0.001
Individual-level	Coefficient (SE)	*p* value	Coefficient (SE)	*p* value	Coefficient (SE)	*p* value	Coefficient (SE)	*p* value	Coefficient (SE)	*p* value	Coefficient (SE)	*p* value	Coefficient (SE)	*p* value
Team job crafting			0.25 (0.19)	0.191	-0.11 (0.22)	0.606	-0.19 (0.24)	0.442						
Crafting for task									-0.05 (0.19)	0.778				
Crafting for respect											-0.07 (0.15)	0.643		
Crafting for information													-0.13 (0.19)	0.502
Ward-level	Coefficient (SE)	*p* value	Coefficient (SE)	*p* value	Coefficient (SE)	*p* value	Coefficient (SE)	*p* value	Coefficient (SE)	*p* value	Coefficient (SE)	*p* value	Coefficient (SE)	*p* value
Team job crafting			-0.19 (0.61)	0.756	-0.08 (0.58)	0.890	-0.13 (0.58)	0.817						
Crafting for task									-0.35 (0.62)	0.568				
Crafting for respect											0.04 (0.43)	0.925		
Crafting for information													0.03 (0.54)	0.962
**Random effects**	Coefficient		Coefficient		Coefficient		Coefficient		Coefficient		Coefficient		Coefficient	
Intercept	0.10		0.11		0.11		0.11		0.10		0.11		0.10	
Residual variance	0.89		0.88		0.90		0.89		0.90		0.90		0.90	

*Nj* the number of wards, *Ni* the number of individuals

^a^Adjusted by age, gender, educational status, marital status, nurse experiences, effort-reward imbalance, T1 work engagement

^b^Adjusted by T1 work engagement

^b^Adjusted by age, gender, educational status, marital status, nurse experiences, effort-reward imbalance, T1 work engagement, job demand, job control, supervisor support, coworker support

### Subgroup analysis

The first subgroup analyses showed no significant association between the total score and any subscale of ward-level team job crafting at T1 and work engagement at T2 and T3 in the higher or lower degree of workplace social capital subgroup and the higher or lower degree of psychological safety subgroup at T1. The results are shown in Supplementary Tables (Tables S1-1 to S2-4).

### Secondary dependent variables results

The results of HLM indicated no significant association between total score and any subscale of ward-level team job crafting at T1 and work performance, psychological distress, and intention to leave at T2 and T3 (Tables 6, 7, 8, 9, 10 and 11, respectively).

**Table 6:** Multilevel association between individual-level and ward-level team job crafting and work performance at T2

	Model 0 (Null model)		Model 1 (Crude model)		Model 2 (adjusted^a^)		Model 3 (adjusted^b^)		Model 3a (adjusted^a^)		Model 3b (adjusted^a^)		Model 3c (adjusted^a^)	
**Fixed effects**	Coefficient (SE)	*p* value	Coefficient (SE)	*p* value	Coefficient (SE)	*p* value	Coefficient (SE)	*p* value	Coefficient (SE)	*p* value	Coefficient (SE)	*p* value	Coefficient (SE)	*p* value
Intercept	5.49 (1.07)	< 0.001	5.50 (1.05)	< 0.001	5.48 (1.05)	< 0.001	5.49 (1.05)	< 0.001	5.48 (1.05)	< 0.001	5.48 (1.05)	< 0.001	5.48 (1.06)	< 0.001
Individual-level	Coefficient (SE)	*p* value							Coefficient (SE)	*p* value	Coefficient (SE)	*p* value	Coefficient (SE)	*p* value
Team job crafting			0.49 (0.24)	0.045	0.42 (0.23)	0.076	0.51 (0.25)	0.042						
Crafting for task									0.36 (0.24)	0.132				
Crafting for respect											0.30 (0.18)	0.105		
Crafting for information													0.31 (0.20)	0.133
Ward-level	Coefficient (SE)	*p* value	Coefficient (SE)	*p* value	Coefficient (SE)	*p* value	Coefficient (SE)	*p* value	Coefficient (SE)	*p* value	Coefficient (SE)	*p* value	Coefficient (SE)	*p* value
Team job crafting			-0.39 (0.87)	0.657	-0.33 (0.85)	0.694	-0.230 (0.84)	0.723						
Crafting for task									-0.39 (0.77)	0.607				
Crafting for respect											-0.15 (0.70)	0.832		
Crafting for information													-0.42 (0.90)	0.639
**Random effects**	Coefficient		Coefficient		Coefficient		Coefficient		Coefficient		Coefficient		Coefficient	
Intercept	0.08		0.07		0.08		0.09		0.09		0.08		0.05	
Residual variance	0.92		0.92		0.91		0.91		0.91		0.92		0.95	
AIC	1680.762		1624.083		1600.147		1608.299		Coefficient (SE)	*p* value	Coefficient (SE)	*p* value	Coefficient (SE)	*p* value

*Nj* the number of wards, *Ni* the number of individuals

^a^Adjusted by age, gender, educational status, marital status, nurse experiences, effort-reward imbalance, T1 work engagement

^b^Adjusted by T1 work engagement

^b^Adjusted by age, gender, educational status, marital status, nurse experiences, effort-reward imbalance, T1 work engagement, job demand, job control, supervisor support, coworker support

**Table 7:** Multilevel association between individual-level and ward-level team job crafting and work performance at T3 (Nj = 30, Ni = 391)

	Model 0 (Null model)		Model 1 (Crude model)		Model 2 (adjusted^a^)		Model 3 (adjusted^b^)		**Model 3a (adjusted** ^**b**^ **)**		**Model 3b (adjusted** ^**b**^ **)**		**Model 3c (adjusted** ^**b**^ **)**	
**Fixed effects**	Coefficient (SE)	*p* value	Coefficient (SE)	*p* value	Coefficient (SE)	*p* value	Coefficient (SE)	*p* value	Coefficient (SE)	*p* value	Coefficient (SE)	*p* value	Coefficient (SE)	*p* value
Intercept	4.88 (1.46)	< 0.001	4.90 (1.45)	< 0.001	4.88 (1.45)	< 0.001	4.88 (1.44)	< 0.001	4.89 (1.44)	< 0.001	4.88 (1.46)	< 0.001	4.88 (1.46)	< 0.001
Individual-level	Coefficient (SE)	*p* value							Coefficient (SE)	*p* value	Coefficient (SE)	*p* value	Coefficient (SE)	*p* value
Team job crafting			0.36 (0.27)	0.194	0.22 (0.30)	0.476	0.19 (0.34)	0.582						
Crafting for task									0.24 (0.28)	0.410				
Crafting for respect											0.20 (0.20)	0.331		
Crafting for information													0.03 (0.28)	0.925
Ward-level	Coefficient (SE)	*p* value	Coefficient (SE)	*p* value	Coefficient (SE)	*p* value	Coefficient (SE)	*p* value	Coefficient (SE)	*p* value	Coefficient (SE)	*p* value	Coefficient (SE)	*p* value
Team job crafting			-0.52 (0.88)	0.559	-0.60 (0.82)	0.461	-0.55 (0.83)	0.511						
Crafting for task									-0.73 (0.94)	0.436				
Crafting for respect											-0.39 (0.63)	0.540		
Crafting for information													-0.42 (0.77)	0.585
**Random effects**	Coefficient		Coefficient		Coefficient		Coefficient		Coefficient		Coefficient		Coefficient	
Intercept	0.10		0.11		0.09		0.09		0.09		0.09		0.09	
Residual variance	0.89		0.89		0.91		0.91		0.90		0.91		0.91	
AIC	1630.408		1575.229		1487.468		1496.230		Coefficient (SE)	*p* value	Coefficient (SE)	*p* value	Coefficient (SE)	*p* value

*Nj* the number of wards, *Ni* the number of individuals

^a^Adjusted by age, gender, educational status, marital status, nurse experiences, effort-reward imbalance, T1 work engagement

^b^Adjusted by T1 work engagement

^b^Adjusted by age, gender, educational status, marital status, nurse experiences, effort-reward imbalance, T1 work engagement, job demand, job control, supervisor support, coworker support

**Table 8:** Multilevel association between individual-level and ward-level team job crafting and psychological distress at T2 (Nj = 30, Ni = 391)

	Model 3a (adjusted^b^)		Model 3b (adjusted^b^)		Model 3c (adjusted^b^)		Model 0 (Null model)		Model 1 (Crude model)		Model 2 (adjusted^a^)		Model 3 (adjusted^b^)	
**Fixed effects**	Coefficient (SE)	*p* value	Coefficient (SE)	*p* value	Coefficient (SE)	*p* value	Coefficient (SE)	*p* value	Coefficient (SE)	*p* value	Coefficient (SE)	*p* value	Coefficient (SE)	*p* value
Intercept	6.53 (3.00)	0.003	6.52 (3.00)	0.003	6.53 (3.00)	0.003	6.69 (3.12)	0.002	6.72 (3.10)	0.001	6.52 (2.99)	0.003	6.54 (2.99)	0.003
Individual-level	Coefficient (SE)	*p* value	Coefficient (SE)	*p* value	Coefficient (SE)	*p* value	Coefficient (SE)	*p* value						
Team job crafting									-1.72 (0.69)	0.013	-0.04 (0.59)	0.941	-0.16 (0.62)	0.802
Crafting for task	-0.21 (0.63)	0.738												
Crafting for respect			-0.03 (0.46)	0.952										
Crafting for information					0.15 (0.52)	0.775								
Ward-level	Coefficient (SE)	*p* value	Coefficient (SE)	*p* value	Coefficient (SE)	*p* value	Coefficient (SE)	*p* value	Coefficient (SE)	*p* value	Coefficient (SE)	*p* value	Coefficient (SE)	*p* value
Team job crafting									0.67 (2.34)	0.802	-0.08 (2.13)	0.969	-0.09 (2.06)	0.965
Crafting for task	1.11 (2.09)	0.596												
Crafting for respect			-0.30 (1.68)	0.858										
Crafting for information					-0.59 (2.18)	0.788								
**Random effects**	Coefficient		Coefficient		Coefficient		Coefficient		Coefficient		Coefficient		Coefficient	
Intercept	0.07		0.07		0.07		0.09		0.08		0.07		0.07	
Residual variance	0.92		0.92		0.92		0.90		0.90		0.92		0.93	

*Nj* the number of wards, *Ni* the number of individuals

^a^Adjusted by age, gender, educational status, marital status, nurse experiences, effort-reward imbalance, T1 work engagement

^b^Adjusted by T1 work engagement

^b^Adjusted by age, gender, educational status, marital status, nurse experiences, effort-reward imbalance, T1 work engagement, job demand, job control, supervisor support, coworker support

**Table 9:** Multilevel association between individual-level and ward-level team job crafting and psychological distress at T3 (Nj = 30, Ni = 391)

	Model 0 (Null model)		Model 1 (Crude model)		Model 2 (adjusted^a^)		Model 3 (adjusted^b^)		Model 3a (adjusted^b^)		Model 3b (adjusted^b^)		Model 3c (adjusted^b^)	
**Fixed effects**	Coefficient (SE)	*p* value	Coefficient (SE)	*p* value	Coefficient (SE)	*p* value	Coefficient (SE)	*p* value	Coefficient (SE)	*p* value	Coefficient (SE)	*p* value	Coefficient (SE)	*p* value
Intercept	6.55 (3.39)	< 0.001	6.58 (3.37)	< 0.001	6.48 (3.34)	< 0.001	6.48 (3.33)	< 0.001	6.46 (3.31)	< 0.001	6.49 (3.36)	< 0.001	6.48 (3.36)	< 0.001
Individual-level	Coefficient (SE)	*p* value							Coefficient (SE)	*p* value	Coefficient (SE)	*p* value	Coefficient (SE)	*p* value
Team job crafting			-1.29 (0.71)	0.071	-0.30 (0.76)	0.697	-0.33 (0.84)	0.696						
Crafting for task									-0.32 (0.65)	0.629				
Crafting for respect											0.03 (0.59)	0.965		
Crafting for information													-0.31 (0.68)	0.647
Ward-level	Coefficient (SE)	*p* value	Coefficient (SE)	*p* value	Coefficient (SE)	*p* value	Coefficient (SE)	*p* value	Coefficient (SE)	*p* value	Coefficient (SE)	*p* value	Coefficient (SE)	*p* value
Team job crafting			0.61 (2.21)	0.783	0.59 (2.07)	0.776	0.60 (2.11)	0.777						
Crafting for task									1.58 (2.27)	0.486				
Crafting for respect											-0.08 (1.57)	0.962		
Crafting for information													0.44 (1.90)	0.819
**Random effects**	Coefficient		Coefficient		Coefficient		Coefficient		Coefficient		Coefficient		Coefficient	
Intercept	0.06		0.06		0.05		0.05		0.05		0.05		0.05	
Residual variance	0.93		0.93		0.95		0.95		0.95		0.95		0.95	
AIC	2294.049		2224.788		2180.044		2185.809		Coefficient (SE)	*p* value	Coefficient (SE)	*p* value	Coefficient (SE)	*p* value

*Nj* the number of wards, *Ni* the number of individuals

^a^Adjusted by age, gender, educational status, marital status, nurse experiences, effort-reward imbalance, T1 work engagement

^b^Adjusted by T1 work engagement

^b^Adjusted by age, gender, educational status, marital status, nurse experiences, effort-reward imbalance, T1 work engagement, job demand, job control, supervisor support, coworker support

**Table 10:** Multilevel association between individual-level and ward-level team job crafting and intention to leave at T2 (Nj = 30, Ni = 391)

	Model 0 (Null model)		Model 1 (Crude model)		Model 2 (adjusted^a^)		Model 3 (adjusted^b^)		Model 3a (adjusted^a^)		Model 3b (adjusted^a^)		Model 3c (adjusted^a^)	
**Fixed effects**	Coefficient (SE)	*p* value	Coefficient (SE)	*p* value	Coefficient (SE)	*p* value	Coefficient (SE)	*p* value	Coefficient (SE)	*p* value	Coefficient (SE)	*p* value	Coefficient (SE)	*p* value
Intercept	2.77 (0.24)	< 0.001	2.78 (0.24)	< 0.001	2.78 (0.23)	< 0.001	2.79 (0.23)	< 0.001	2.78 (0.23)	< 0.001	2.78 (0.23)	< 0.001	2.78 (0.23)	< 0.001
Individual-level	Coefficient (SE)	*p* value							Coefficient (SE)	*p* value	Coefficient (SE)	*p* value	Coefficient (SE)	*p* value
Team job crafting			-0.16 (0.07)	0.017	-0.02 (0.06)	0.764	0.01 (0.06)	0.908						
Crafting for task									-0.01 (0.06)	0.925				
Crafting for respect											-0.02 (0.04)	0.601		
Crafting for information													-0.01 (0.05)	0.805
Ward-level	Coefficient (SE)	*p* value	Coefficient (SE)	*p* value	Coefficient (SE)	*p* value	Coefficient (SE)	*p* value	Coefficient (SE)	*p* value	Coefficient (SE)	*p* value	Coefficient (SE)	*p* value
Team job crafting			-0.06 (0.21)	0.775	-0.02 (0.18)	0.930	-0.01 (0.19)	0.973						
Crafting for task									0.13 (0.18)	0.467				
Crafting for respect											-0.03 (0.14)	0.836		
Crafting for information													-0.05 (0.18)	0.795
**Random effects**	Coefficient		Coefficient		Coefficient		Coefficient		Coefficient		Coefficient		Coefficient	
Intercept	0.16		0.15		0.08		0.08		0.09		0.08		0.08	
Residual variance	0.84		0.85		0.91		0.91		0.91		0.92		0.92	
AIC	659.094		631.155		621.645		640.845		Coefficient (SE)	*p* value	Coefficient (SE)	*p* value	Coefficient (SE)	*p* value

*Nj* the number of wards, *Ni* the number of individuals

^a^Adjusted by age, gender, educational status, marital status, nurse experiences, effort-reward imbalance, T1 work engagement

^b^Adjusted by T1 work engagement

^b^Adjusted by age, gender, educational status, marital status, nurse experiences, effort-reward imbalance, T1 work engagement, job demand, job control, supervisor support, coworker support

**Table 11:** Multilevel association between individual-level and ward-level team job crafting and intention to leave at T3 (Nj = 30, Ni = 391)

	Model 0 (Null model)		Model 1 (Crude model)		Model 2 (adjusted^a^)		Model 3 (adjusted^b^)		Model 3a (adjusted^b^)		Model 3b (adjusted^b^)		Model 3c (adjusted^b^)	
**Fixed effects**	Coefficient (SE)	*p* value	Coefficient (SE)	*p* value	Coefficient (SE)	*p* value	Coefficient (SE)	*p* value	Coefficient (SE)	*p* value	Coefficient (SE)	*p* value	Coefficient (SE)	*p* value
Intercept	2.75 (0.33)	< 0.001	2.75 (0.33)	< 0.001	2.75 (0.33)	< 0.001	2.75 (0.33)	< 0.001	2.75 (0.33)	< 0.001	2.75 (0.33)	< 0.001	2.76 (0.33)	< 0.001
Individual-level	Coefficient (SE)	*p* value							Coefficient (SE)	*p* value	Coefficient (SE)	*p* value	Coefficient (SE)	*p* value
Team job crafting			-0.15 (0.09)	0.101	-0.02 (0.08)	0.755	-0.01 (0.09)	0.929						
Crafting for task									-0.01 (0.07)	0.909				
Crafting for respect											-0.03 (0.06)	0.571		
Crafting for information													-0.02 (0.07)	0.808
Ward-level	Coefficient (SE)	*p* value	Coefficient (SE)	*p* value	Coefficient (SE)	*p* value	Coefficient (SE)	*p* value	Coefficient (SE)	*p* value	Coefficient (SE)	*p* value	Coefficient (SE)	*p* value
Team job crafting			-0.04 (0.25)	0.866	0.003 (0.23)	0.987	0.01 (0.23)	0.976						
Crafting for task									0.14 (0.25)	0.585				
Crafting for respect											-0.004 (0.18)	0.983		
Crafting for information													-0.07 (0.21)	0.754
**Random effects**	Coefficient		Coefficient		Coefficient		Coefficient		Coefficient		Coefficient		Coefficient	
Intercept	0.16		0.16		0.12		0.11		0.12		0.12		0.11	
Residual variance	0.82		0.82		0.87		0.88		0.87		0.87		0.88	
AIC	712.017		700.818		721.781		742.125		Coefficient (SE)	*p* value	Coefficient (SE)	*p* value	Coefficient (SE)	*p* value

*Nj* the number of wards, *Ni* the number of individuals

^a^Adjusted by age, gender, educational status, marital status, nurse experiences, effort-reward imbalance, T1 work engagement

^b^Adjusted by T1 work engagement

^b^Adjusted by age, gender, educational status, marital status, nurse experiences, effort-reward imbalance, T1 work engagement, job demand, job control, supervisor support, coworker support

### Associations between changes in team job crafting and changes in work engagement

Additional analyses were conducted to examine the association between the ward-level change of team job crafting (T2 – T1 and T3 – T1, respectively) and the individual-level change of work engagement (T2 – T1 and T3 – T1, respectively) (see Tables 12 and 13). The ward-level change in the total score of team job crafting from T1 to T2 and from T1 to T3 was not significantly associated with the individual-level change in work engagement. The ward-level change in crafting for the task considering the team's growth from T1 to T3, which is one of the subscales of team job crafting, was significantly and positively associated with the individual-level change in work engagement from T1 to T3 (*Coefficient* = 0.65 [SE = 0.29], *p* = 0.024). The individual-level changes in the total team job crafting score, crafting for the task considering the team's growth, and crafting for smooth information sharing of individual-level team job crafting from T1 to T2 were significantly and positively associated with the change of work engagement from T1 to T2. The individual-level changes in the total score and each subscale of individual-level team job crafting from T1 to T3 was also significantly and positively associated with the change in work engagement from T1 to T3. There was no significant association between the ward-level change of team job crafting (T2 – T1) and the individual-level change of work engagement (T3 – T2) (Table 14).

**Table 12:** Multilevel association between change of team job crafting (T2-T1) and change of work engagement (T2-T1) (Nj = 23, Ni = 248)

	Model 0 (Null model)		Model 1 (Crude model)		Model 2 (adjusted^a^)		Model 3 (adjusted^b^)	
**Fixed effects**	Coefficient (SE)	*p* value	Coefficient (SE)	*p* value	Coefficient (SE)	*p* value	Coefficient (SE)	*p* value
Intercept	0.003 (0.07)	0.963	-0.002 (0.06)	0.969	-0.01 (0.06)	0.831	-0.02 (0.07)	0.766
Individual-level changes	Coefficient (SE)	*p* value	Coefficient (SE)	*p* value	Coefficient (SE)	*p* value	Coefficient (SE)	*p* value
Team job crafting (T2-T1)			0.25 (0.08)	0.001	0.25 (0.08)	0.001	0.25 (0.08)	0.001
Crafting for task (T2-T1)								
Crafting for respect (T2-T1)								
Crafting for information(T2-T1)								
Ward-level changes	Coefficient (SE)	*p* value	Coefficient (SE)	*p* value	Coefficient (SE)	*p* value	Coefficient (SE)	*p* value
Team job crafting (T2-T1)			0.56 (0.36)	0.117	0.61 (0.36)	0.095	0.59 (0.38)	0.116
Crafting for task (T2-T1)								
Crafting for respect (T2-T1)								
Crafting for information(T2-T1)								
**Random effects**	Coefficient		Coefficient		Coefficient		Coefficient	
Intercept (Ward)	0.17		0.16		0.16		0.18	
Residual variance	0.83		0.84		0.84		0.82	
AIC	441.303		432.939		461.569		482.485	

*Nj* the number of wards, *Ni* the number of individuals

^a^Adjusted by age, gender, educational status, marital status, nurse experiences, effort-reward imbalance

^b^Adjusted by age, gender, educational status, marital status, nurse experiences, effort-reward imbalance, job demand, job control, supervisor support, coworker support

**Table 13:** Multilevel association between change of team job crafting (T3-T1) and change of work engagement (T3-T1) (Nj = 21, Ni = 175)

	Model 0 (Null model)		Model 1 (Crude model)		Model 2 (adjusted^a^)		Model 3 (adjusted^b^)		Model 2a (adjusted^b^)		Model 2b (adjusted^b^)		Model 2c (adjusted^b^)	
**Fixed effects**	Coefficient (SE)	*p* value	Coefficient (SE)	*p* value	Coefficient (SE)	*p* value	Coefficient (SE)	*p* value	Coefficient (SE)	*p* value	Coefficient (SE)	*p* value	Coefficient (SE)	*p* value
Intercept	-0.04 (0.06)	0.504	-0.05 (0.05)	0.291	-0.06 (0.05)	0.231	-0.06 (0.05)	0.232	-0.05 (0.05)	0.299	-0.07 (0.06)	0.249	-0.05 (0.06)	0.403
Individual-level changes	Coefficient (SE)	*p* value	Coefficient (SE)	*p* value	Coefficient (SE)	*p* value	Coefficient (SE)	*p* value	Coefficient (SE)	*p* value	Coefficient (SE)	*p* value	Coefficient (SE)	*p* value
Team job crafting (T3-T1)			0.51 (0.11)	< 0.001	0.55 (0.11)	< 0.001	0.55 (0.11)	< 0.001						
Crafting for task (T3-T1)									0.34 (0.10)	< 0.001				
Crafting for respect (T3-T1)											0.30 (0.09)	0.002		
Crafting for information(T3-T1)													0.41 (0.09)	< 0.001
Ward-level changes	Coefficient (SE)	*p* value	Coefficient (SE)	*p* value	Coefficient (SE)	*p* value	Coefficient (SE)	*p* value	Coefficient (SE)	*p* value	Coefficient (SE)	*p* value	Coefficient (SE)	*p* value
Team job crafting (T3-T1)			0.40 (0.31)	0.191	0.40 (0.31)	0.202	0.38 (0.31)	0.253						
Crafting for task (T3-T1)									0.65 (0.29)	0.024				
Crafting for respect (T3-T1)											0.36 (0.26)	0.179		
Crafting for information(T3-T1)													0.13 (0.30)	0.679
**Random effects**	Coefficient		Coefficient		Coefficient		Coefficient		Coefficient		Coefficient		Coefficient	
Intercept (Ward)	0.04		0.01		0.01		0.00		0.00		0.02		0.05	
Residual variance	0.96		1.00		1.00		1.00		1.00		0.98		0.95	
AIC	374.018		350.321		372.208		389.134		Coefficient (SE)	*p* value	Coefficient (SE)	*p* value	Coefficient (SE)	*p* value

*Nj* the number of wards, *Ni* the number of individuals

^a^Adjusted by age, gender, educational status, marital status, nurse experiences, effort-reward imbalance

^b^Adjusted by T1 work engagement

^b^Adjusted by age, gender, educational status, marital status, nurse experiences, effort-reward imbalance, T1 work engagement, job demand, job control, supervisor support, coworker support

**Table 14:** Multilevel association between change of team job crafting (T2-T1) and change of work engagement (T3-T2) (Nj = 21, Ni = 248)

	Model 0 (Null model)		Model 1 (Crude model)		Model 2 (adjusted^a^)		Model 3 (adjusted^b^)		Model 2a (adjusted^b^)		Model 2b (adjuste^b^)		Model 2c (adjusted^b^)	
**Fixed effects**	Coefficient (SE)	*p* value	Coefficient (SE)	*p* value	Coefficient (SE)	*p* value	Coefficient (SE)	*p* value	Coefficient (SE)	*p* value	Coefficient (SE)	*p* value	Coefficient (SE)	*p* value
Intercept	-0.05 (0.07)	0.467	-0.05 (0.07)	0.460	-0.06 (0.07)	0.434	-0.06 (0.07)	0.408	-0.06 (0.07)	0.421	-0.06 (0.07)	0.410	-0.06 (0.07)	0.409
Individual-level changes	Coefficient (SE)	*p* value	Coefficient (SE)	*p* value	Coefficient (SE)	*p* value	Coefficient (SE)	*p* value	Coefficient (SE)	*p* value	Coefficient (SE)	*p* value	Coefficient (SE)	*p* value
Team job crafting (T2-T1)			0.06 (0.20)	0.777	0.06 (0.19)	0.770	0.06 (0.19)	0.771						
Crafting for task (T2-T1)									0.05 (0.13)	0.687				
Crafting for respect (T2-T1)											0.001 (0.09)	0.986		
Crafting for information(T2-T1)													0.08 (0.21)	0.710
Ward-level changes	Coefficient (SE)	*p* value	Coefficient (SE)	*p* value	Coefficient (SE)	*p* value	Coefficient (SE)	*p* value	Coefficient (SE)	*p* value	Coefficient (SE)	*p* value	Coefficient (SE)	*p* value
Team job crafting (T2-T1)			0.17 (0.43)	0.695	0.27 (0.46)	0.557	0.22 (0.46)	0.629						
Crafting for task (T2-T1)									0.14 (0.34)	0.687				
Crafting for respect (T2-T1)											0.15 (0.29)	0.609		
Crafting for information(T2-T1)													0.11 (0.43)	0.803
**Random effects**	Coefficient		Coefficient		Coefficient		Coefficient		Coefficient		Coefficient		Coefficient	
Intercept (Ward)	0.03		0.03		0.02		0.02		0.02		0.02		0.02	
Residual variance	0.99		0.99		0.99		0.99		0.99		0.99		0.99	
AIC	466.481		473.771		499.121		519.594		Coefficient (SE)	*p* value	Coefficient (SE)	*p* value	Coefficient (SE)	*p* value

*Nj* the number of wards, *Ni* the number of individuals

^a^Adjusted by age, gender, educational status, marital status, nurse experiences, effort-reward imbalance

^b^Adjusted by age, gender, educational status, marital status, nurse experiences, job demand, job control, supervisor support, coworker support, effort-reward imbalance

## Discussion

Contrary to the hypotheses, this longitudinal study found no significant multilevel association between ward-level team job crafting at baseline and individual-level work engagement at three-month or six-month follow-ups among nurses. The result was same for the association between each subscale of ward-level team job crafting and work engagement. Subgroup analyses showed that there was no significant difference in the longitudinal relationship between the total and any subscale score of ward-level team job crafting and work engagement in the degree of workplace social capital and psychological safety. Ward-level team job crafting had no significant longitudinal associations with work performance, psychological distress, or intention to leave. The additional analyses for testing the difference found that ward-level change of "crafting for the task considering the team's growth," one of the subscales of team job crafting, from T1 to T3 was significantly and positively associated with an individual-level change of work engagement from T1 to T3. The change in the individual-level team job crafting was significantly and positively related to the change in work engagement from T1 to T2 and from T1 to T3, respectively.

The main hypothesis of the association between ward-level team job crafting at T1 and individual-level work engagement at T2 and T3 was not supported. These results were inconsistent with previous cross-sectional and longitudinal studies [24, 27–30]. One possible explanation may be the difference in the follow-up period. A previous study among clinicians including nurses investigated its longitudinal association with a one-month follow-up [30], whereas the current study investigated three-month and six-month follow-ups. Our results suggested that the ward-level behavior of team job crafting might have a synchronous association with work engagement, while the association could not exist over three-months follow-up. According to a meta-analysis that examined the longitudinal association between individual-level job crafting and work engagement among workers, longitudinal observational studies with more than three-month follow-ups found no significant association [68]. Job crafting involves a temporary requirement to achieve short-term work goals, such as enhancing a particular work process and making decisions by team members on which job resources are needed to mobilize [20, 24, 69]. As a result, the benefit of team job crafting is likely to be temporary. Also, since work engagement reflects a highly stable mental state according to previous studies [70], work engagement may remain stable without team members’ continuous crafting behavior for job resources.

Considering cross-sectional evidence in previous research, the association between team job crafting and work engagement found in prior studies may have been causally reversed, at least in part. A concept paper suggested that engaged workers are more likely to increase their job resources, that is to say, are more likely to craft their jobs [71]. They also said that engaged workers tend to try to be actively involved in organizational matters [71]. Therefore, team job crafting might be observed in a workplace where team members' work engagement is already high in the previous studies.

The difference in the systems and culture of Japanese hospitals may also have influenced the results because the previous studies among nurses were conducted in China and Vietnam. For example, Japan has the most hospital beds and a low nurse-to-patient ratio [72]. Due to a higher workload, Japanese nurses work more involuntary overtime than in other countries [73–75]. It is possible that nurses who participated in this study were so occupied with their daily work that doing team job crafting did not improve their work engagement, but made them feel like they were forced to do it by other team members.

Another possible reason for the null result may be that other non-negligible confounding variables could have distorted the results. First, the impact of COVID-19 on work engagement might be much larger. In Japan, the sixth wave of the COVID-19 pandemic occurred from January to April 2022 (during the T2 survey), and the seventh wave occurred from June to September (during the T3 survey) [76]. During the pandemic's peak, nurses perceived a high emotional work and a heavy workload [77], and the COVID-19 crisis was negatively related to work engagement among front-line health workers [78]. Thus, the spread of COVID-19 may have resulted in lower work engagement, regardless of the baseline level of team job crafting. The second possible confounding factor might be organizational change. At Japanese hospitals, many nurses often retire, and new nurses are hired from the end of March to the beginning of April, resulting in major changes in the composition of ward members. A previous study found that adverse changes in the work environment were related to the job distress of nurses [79]. They discussed that workplace restructuring goes hand-in-hand with constant training of new members, who could be in high demand and high job stress for nurses who remain on the ward with few human resources [79]. Therefore, organizational change may impact low work engagement irrespective of baseline team job crafting.

Another possibility is that team job crafting at T1 increased work engagement at T1 but did not affect work engagement over time. In exploring this idea, the present study observed a moderate ward-level correlation between team job crafting and work engagement at T1 (0.25). The individual-level correlation coefficient between work engagement at T1 and work engagement at T2 and T3 were 0.83 and 0.79, respectively. While team job crafting at T1 may not be associated with the change of work engagement over time, it may be associated with higher levels of work engagement at T1-T3. Future longitudinal studies could consider this possibility for their analyses.

The ICCs for team job crafting were low (0.03 to 0.12), a result that was inconsistent with a previous study among nurses [22]. A possible explanation is that the low response rate of the current study might cause the low ICC of team job crafting. If participants with relatively unengaged team job crafting behavior did not respond to the questionnaire, team job crafting scores between the wards would have stayed the same, and within-group similarities could not have been detected. The low response rate allowed a low number of nurses to be included in the ward-level analysis. In some wards, only a small number of nurses provided their data, which limited the variable as an indicator for ward-level team job crafting. Also, the inclusion criterion of wards with at least two nurses responding may have been too few considering the representativeness of the population. Other possibilities include that the team job crafting scale could not capture the ward-level variance of team job crafting. Another is that the team job crafting may not be a collective construct, while it is supposed to be so in the proposed theory [20, 21]. If so, even if they are in the same wards, individual nurses would have different perspectives on the degree of crafting their jobs. This might require a reconsideration of the concept of team job crafting.

Moreover, the ICCs for work engagement (0.01 to 0.05) and the design effect (1.60) were low. Previous studies among healthcare workers, including nurses, have also found ICCs ranging from 0.05 to 0.09, indicating the variance of work engagement is less likely to be shared by the group and more likely to be explained at the individual level [56, 80]. If work engagement is a variable provided by individual-level factors, and there are few group-level effects on work engagement, the model of the hypothesis assuming group-level team job crafting association may have low plausibility. Accordingly, when investigating the multi-level impact on work engagement among healthcare workers, it is necessary to consider that the contextual effect of work engagement may be small.

The second hypothesis proposing that the degree of association between ward-level team job crafting at T1 and individual-level work engagement at T2 and T3 was greater in the wards with higher degrees of workplace social capital and psychological safety was not supported. The present study found no association between ward-level team job crafting and work engagement in either subgroup classified by the degree of organizational climate. Although team job crafting behavior is more likely to occur when working in a better organizational climate among general workers [34, 81–83] the degree of organizational climate may not make a difference in understanding and accepting team job crafting behaviors. It may not be involved in the longitudinal relationship between team job crafting and work engagement.

The third hypothesis regarding a positive association between ward-level team job crafting at T1 with work performance, psychological distress, and intention to leave at T2 and T3 was not supported. Similar to the reasons why H1 was not supported, the effects of team job crafting may not maintain for a long period to improve work performance and decrease psychological distress and intention to leave among nurses.

The fourth hypothesis for a positive association between the ward-level change of team job crafting scores (from T1 to T2 and T3) with the individual-level change of work engagement scores (from T1 to T2 and T3) was partially supported. An increase in "crafting for the task considering the team's growth" through six months was associated with work engagement increases through six months. While ward-level team job crafting behavior at a single point in time does not predict future work engagement, an increased degree of crafting for their growth may be related to an increase in individual's work engagement. This subscale represents a concept similar to collaborative job crafting and another theory that focuses on task crafting and increasing structural job resources [22]. The fact that increasing a task-focused type of team job crafting is positively associated with increased work engagement is consistent with the results of previous studies among both healthcare and non-healthcare workers [24, 27, 28, 30]. Also, an individual-level increase in team job crafting was significantly and positively related to the increase in work engagement from T1 to T2 and from T1 to T3, respectively. This may indicate that nurses who had a positive attitude toward team job crafting activities in a ward increased their work engagement. It is also possible that nurses who participated more in these activities increased work engagement. Better informing team members regarding team job crafting activities in a group and facilitating the participation of team members in team job crafting may be more important as drivers of work engagement than an average level of team job crafting in a group. Such dynamic psychological processes related to team job crafting should be investigated further.

### Limitation

There are several limitations to this study. First, the number of participants and wards included in the analysis met only about 20% of the expected sample size, and response rates varied by ward. The low sample size may cause very low statistical power in the analyses (1-β = 0.20) and lead to type 1 and type 2 errors. Multilevel modeling may also not be possible due to the small number of participating wards. Additionally, the low response rate of the participants (18.6%) and the low follow-up rates (60.7% for the T2 survey and 45.2% for the T3 survey, respectively) can cause selection bias. If the participants with low team job crafting and low work engagement did not complete the questionnaire, the associations might have been underestimation. Furthermore, because ICCs for work engagement were very low, perhaps because of the low response rate within the wards, there was less opportunity to examine the between-level association in the multilevel model. Also, we included all the data from those respondents who took part in the baseline for the analyses. If there were some differences in characteristics between those who remained in the study and those who dropped out, the generalizability of the results would be limited. Second, all individual- and ward-level variables were measured using self-report questionnaires. Especially, work performance was assessed by the respondents themselves and the scale did not evaluate content validity with objective indicators such as supervisor’s ratings of work performance. Measured values could contain information bias and measurement errors. Third, the results may be distorted by confounding variables that this study could not consider, such as other types of job stressors and environmental determinants outside the workplace. Fourth, limited causal inferences could be made when testing the fourth hypothesis, as there was no temporal separation between the independent and dependent variables. Nevertheless, analysis of relationships between concurrent change processes has been suggested for theoretical operationalization [41] and is consistent with previous reports about work engagement [84]. Finally, hospitals were selected from a limited area in Japan by snowball sampling methods, and nurses who were interested in the study were asked to respond to the questionnaire. However, the use of snowball sampling may have introduced bias because it is possible the sample wards were supportive and had a more positive attitude toward team job crafting, limiting the generalizability of the results. High-quality investigations, such as the random sampling method, must be conducted to solve these limitations.

### Implication

The current study investigated the multilevel longitudinal associations between ward-level team job crafting and individual-level work engagement and other work-related outcomes among nurses. The results showed no significant longitudinal association. While team job crafting has been put forward as a way to promote work engagement, this study implies that there is no long-term association. Because most studies have only focused on the positive aspects of team job crafting, future research should consider the disadvantages of team job crafting as well. However, the increase in ward-level crafting for the task considering team growth may be related to an individual-level increase in work engagement. In hospital wards, nursing managers setting up an environment to encourage crafting task boundaries for team members’ growth may contribute to increasing nurses' work engagement. Furthermore, future research should investigate the relationship between team job crafting and other organizational outcomes such as organizational commitment, psychological safety, and organizational citizenship behavior.

## Conclusions

This study investigated the association between ward-level team job crafting and individual-level work engagement among Japanese nurses using a multilevel longitudinal design. The current study found that ward-level team job crafting at baseline was not associated with positive individual outcomes such as work engagement and work performance and negative individual outcomes such as psychological distress and intention to leave at three-month and six-month follow-ups. The impact of ward-level team job crafting on work engagement may not exist in a longer-term follow-up. The ward-level increase in the crafting for a task considering team growth may be related to an individual-level increase in work engagement. This study has practical implications for designing team job crafting interventions, including an approach for team growth to increase nurses' work engagement.

### Supplementary Information

**Additional file 1.** STROBE Statement—checklist of items that should be included in reports of observational studies.

**Additional file 2: Appendix 2. Supplementary Tables.** Table S1-1. Multilevel association between individual-level and ward-level team job crafting and work engagement among workplace social capital [high] ward at T2 (Nj= 17, Ni= 196). Table S1-2. Multilevel association between individual-level and ward-level team job crafting and work engagement among workplace social capital [low] ward at T2 (Nj= 13, Ni= 195). Table S1-3. Multilevel association between individual-level and ward-level team job crafting and work engagement among workplace social capital [high] ward at T3 (Nj= 17, Ni= 196). Table S1-4. Multilevel association between individual-level and ward-level team job crafting and work engagement among workplace social capital [low] ward at T3 (Nj= 13, Ni= 195). Table S2-1. Multilevel association between individual-level and ward-level team job crafting and work engagement among psychological safety [high] ward at T2 (Nj= 17, Ni= 180). Table S2-2. Multilevel association between individual-level and ward-level team job crafting and work engagement among psychological safety [low] ward at T2 (Nj= 13, Ni= 211). Table S2-3. Multilevel association between individual-level and ward-level team job crafting and work engagement among psychological safety [high] ward at T3 (Nj= 17, Ni= 180). Table S2-4. Multilevel association between individual-level and ward-level team job crafting and work engagement among psychological safety [low] ward at T3 (Nj= 13, Ni= 211).

## Data Availability

The datasets used and/or analysed during the current study are available from the corresponding author on reasonable request.
