# Supplementary material for: The association between team job crafting and work engagement among nurses: a prospective cohort study
Source: BMC Psychol. 2024 Feb 9;12:66. doi: 10.1186/s40359-024-01538-7 (PMC10854162; doi:10.1186/s40359-024-01538-7)
Supplement: Supplementary file 1 — Additional file 1. STROBE Statement—checklist of items that should be included in reports of observational studies. [file 40359_2024_1538_MOESM1_ESM.docx]

STROBE Statement—checklist of items that should be included in reports of observational studies

|  | Item No. | Recommendation | Page  No. | Relevant text from manuscript |
| --- | --- | --- | --- | --- |
| **Title and abstract** | 1 | (*a*) Indicate the study’s design with a commonly used term in the title or the abstract | p.1 | The association between team job crafting and work engagement among nurses:  a prospective cohort study |
|  |  | (*b*) Provide in the abstract an informative and balanced summary of what was done and what was found | p.1-2 | Abstract |
| Introduction | | | |  |
| Background/rationale | 2 | Explain the scientific background and rationale for the investigation being reported | p.3-8 |  |
| Objectives | 3 | State specific objectives, including any prespecified hypotheses | p.7-9 | - This study aimed to investigate the longitudinal association between ward-level team job crafting at baseline and individual-level work engagement at three-month and six-month follow-ups among Japanese nurses.  - Hypotheses |
| Methods | | | |  |
| Study design | 4 | Present key elements of study design early in the paper | p.10 | Study design |
| Setting | 5 | Describe the setting, locations, and relevant dates, including periods of recruitment, exposure, follow-up, and data collection | p.10 | Study design |
| Participants | 6 | (*a*) *Cohort study*—Give the eligibility criteria, and the sources and methods of selection of participants. Describe methods of follow-up  *Case-control study*—Give the eligibility criteria, and the sources and methods of case ascertainment and control selection. Give the rationale for the choice of cases and controls  *Cross-sectional study*—Give the eligibility criteria, and the sources and methods of selection of participants | p.10-11 | Participants |
|  |  | (*b*) *Cohort study*—For matched studies, give matching criteria and number of exposed and unexposed  *Case-control study*—For matched studies, give matching criteria and the number of controls per case | N/A |  |
| Variables | 7 | Clearly define all outcomes, exposures, predictors, potential confounders, and effect modifiers. Give diagnostic criteria, if applicable | p.11-16 | Variables |
| Data sources/ measurement | 8* | For each variable of interest, give sources of data and details of methods of assessment (measurement). Describe comparability of assessment methods if there is more than one group | p.11-16 | Variables |
| Bias | 9 | Describe any efforts to address potential sources of bias | p.11-16 | - Variables  - Confounding variables |
| Study size | 10 | Explain how the study size was arrived at | p.16-17 | Sample size calculation |

Continued on next page

| Quantitative variables | 11 | Explain how quantitative variables were handled in the analyses. If applicable, describe which groupings were chosen and why | p.17-20 | - Statistical analyses  - Subgroup analyses |
| --- | --- | --- | --- | --- |
| Statistical methods | 12 | (*a*) Describe all statistical methods, including those used to control for confounding | p.17-20 | - Statistical analyses  - Subgroup analyses |
|  |  | (*b*) Describe any methods used to examine subgroups and interactions | p.20 | Subgroup analyses |
|  |  | (*c*) Explain how missing data were addressed | p.19 | Using the multiple imputation approach for parameter estimation (56,57), the wards and nurses with partially missing values, except for the ward data, or who dropped out of the study during follow-up were included in the evaluated model. |
|  |  | (*d*) *Cohort study*—If applicable, explain how loss to follow-up was addressed  *Case-control study*—If applicable, explain how matching of cases and controls was addressed  *Cross-sectional study*—If applicable, describe analytical methods taking account of sampling strategy | p.21-22 | Nurses who responded T1 survey were included in the analyses even if they dropped out at T2 or T3 survey. |
|  |  | (*e*) Describe any sensitivity analyses | N/A |  |
| Results | | | | |
| Participants | 13* | (a) Report numbers of individuals at each stage of study—eg numbers potentially eligible, examined for eligibility, confirmed eligible, included in the study, completing follow-up, and analysed | p.21-22 | Participants |
|  |  | (b) Give reasons for non-participation at each stage | p.21-22 | Participants |
|  |  | (c) Consider use of a flow diagram | Figure 1 |  |
| Descriptive data | 14* | (a) Give characteristics of study participants (eg demographic, clinical, social) and information on exposures and potential confounders | p.22 | Characteristics of participants |
|  |  | (b) Indicate number of participants with missing data for each variable of interest | p.21-22 | Participants |
|  |  | (c) *Cohort study*—Summarise follow-up time (eg, average and total amount) | p.21-22 | Participants |
| Outcome data | 15* | *Cohort study*—Report numbers of outcome events or summary measures over time | p.22, Table 2 | Table 2 presents the means and SDs of the independent variables at baseline and dependent variables at baseline, 3-month, and 6-month follow-up. |
|  |  | *Case-control study—*Report numbers in each exposure category, or summary measures of exposure | N/A |  |
|  |  | *Cross-sectional study—*Report numbers of outcome events or summary measures | N/A |  |
| Main results | 16 | (*a*) Give unadjusted estimates and, if applicable, confounder-adjusted estimates and their precision (eg, 95% confidence interval). Make clear which confounders were adjusted for and why they were included | p.23,  Tables 4-1 and 4-2 | Longitudinal association between team job crafting and work engagement. |
|  |  | (*b*) Report category boundaries when continuous variables were categorized | N/A |  |
|  |  | (*c*) If relevant, consider translating estimates of relative risk into absolute risk for a meaningful time period | N/A |  |

Continued on next page

| Other analyses | 17 | Report other analyses done—eg analyses of subgroups and interactions, and sensitivity analyses | p.23-24,  Tables 5-1 to 8-2,  Tables S1-1 to S2-4 | - Subgroup analyses  - Secondary dependent variables results  - Associations between changes in team job crafting and changes in work engagement |
| --- | --- | --- | --- | --- |
| Discussion | | | | |
| Key results | 18 | Summarise key results with reference to study objectives | p.25-26 | The first paragraph of the discussion section. |
| Limitations | 19 | Discuss limitations of the study, taking into account sources of potential bias or imprecision. Discuss both direction and magnitude of any potential bias | p.31-32 | Limitation |
| Interpretation | 20 | Give a cautious overall interpretation of results considering objectives, limitations, multiplicity of analyses, results from similar studies, and other relevant evidence | p.32-33 | Implication, conclusion |
| Generalisability | 21 | Discuss the generalisability (external validity) of the study results | p.32 | Finally, because the samples were extracted from a restricted area in Japan by snowball sampling, the generalizability of the results is restricted. |
| Other information | |  | | |
| Funding | 22 | Give the source of funding and the role of the funders for the present study and, if applicable, for the original study on which the present article is based | Declarations |  |

*Give information separately for cases and controls in case-control studies and, if applicable, for exposed and unexposed groups in cohort and cross-sectional studies.

**Note:** An Explanation and Elaboration article discusses each checklist item and gives methodological background and published examples of transparent reporting. The STROBE checklist is best used in conjunction with this article (freely available on the Web sites of PLoS Medicine at http://www.plosmedicine.org/, Annals of Internal Medicine at http://www.annals.org/, and Epidemiology at http://www.epidem.com/). Information on the STROBE Initiative is available at www.strobe-statement.org.
