# Supplementary material for: The association between team job crafting and work engagement among nurses: a prospective cohort study
Source: BMC Psychol. 2024 Feb 9;12:66. doi: 10.1186/s40359-024-01538-7 (PMC10854162; doi:10.1186/s40359-024-01538-7)
Supplement: Supplementary file 2 — Additional file 2: Appendix 2. Supplementary Tables. Table S1-1. Multilevel association between individual-level and ward-level team job crafting and work engagement among workplace social capital [high] ward at T2 (Nj= 17, Ni= 196). Table S1-2. Multilevel association between individual-level and ward-level team job crafting and work engagement among workplace social capital [low] ward at T2 (Nj= 13, Ni= 195). Table S1-3. Multilevel association between individual-level and ward-level team job crafting and work engagement among workplace social capital [high] ward at T3 (Nj= 17, Ni= 196). Table S1-4. Multilevel association between individual-level and ward-level team job crafting and work engagement among workplace social capital [low] ward at T3 (Nj= 13, Ni= 195). Table S2-1. Multilevel association between individual-level and ward-level team job crafting and work engagement among psychological safety [high] ward at T2 (Nj= 17, Ni= 180). Table S2-2. Multilevel association between individual-level and ward-level team job crafting and work engagement among psychological safety [low] ward at T2 (Nj= 13, Ni= 211). Table S2-3. Multilevel association between individual-level and ward-level team job crafting and work engagement among psychological safety [high] ward at T3 (Nj= 17, Ni= 180). Table S2-4. Multilevel association between individual-level and ward-level team job crafting and work engagement among psychological safety [low] ward at T3 (Nj= 13, Ni= 211). [file 40359_2024_1538_MOESM2_ESM.docx]

# **Appendix 2. Supplementary Tables.**

Table S1-1. Multilevel association between individual-level and ward-level team job crafting and work engagement among workplace social capital [high] ward at T2 (Nj= 17, Ni= 196)

|  | Model 0 (Null model) | | Model 1 (Crude model) | | Model 2 (adjusted^a^) | | Model 3 (adjusted^b^) | |
| --- | --- | --- | --- | --- | --- | --- | --- | --- |
| Fixed effects | Coefficient (SE) | p value | Coefficient (SE) | p value | Coefficient (SE) | p value | Coefficient (SE) | p value |
| Intercept | 2.51 (0.43) | <0.001 | 2.54 (0.51) | <0.001 | 2.48 (0.51) | <0.001 | 2.43 (0.51) | <0.001 |
| Individual-level | Coefficient (SE) | p value | Coefficient (SE) | p value | Coefficient (SE) | p value | Coefficient (SE) | p value |
| Team job crafting |  |  | 0.38 (0.15) | 0.015 | -0.05 (0.14) | 0.717 | -0.16 (0.15) | 0.281 |
| Crafting for task |  |  |  |  |  |  |  |  |
| Crafting for respect |  |  |  |  |  |  |  |  |
| Crafting for information |  |  |  |  |  |  |  |  |
| Ward-level | Coefficient (SE) | p value | Coefficient (SE) | p value | Coefficient (SE) | p value | Coefficient (SE) | p value |
| Team job crafting |  |  | -0.44 (0.74) | 0.557 | -0.12 (0.69) | 0.862 | -0.05 (0.59) | 0.939 |
| Crafting for task |  |  |  |  |  |  |  |  |
| Crafting for respect |  |  |  |  |  |  |  |  |
| Crafting for information |  |  |  |  |  |  |  |  |
| Random effects | Coefficient |  | Coefficient |  | Coefficient |  | Coefficient |  |
| Intercept | 0.10 |  | 0.08 |  | 0.12 |  | 0.10 |  |
| Residual variance | 0.90 |  | 0.92 |  | 0.87 |  | 0.89 |  |
| AIC | 673.446 |  | 663.728 |  | 627.805 |  | 637.008 |  |

|  | Model 3a (adjusted^b^) | | Model 3b (adjusted^b^) | | Model 3c (adjusted^b^) | |
| --- | --- | --- | --- | --- | --- | --- |
| Fixed effects | Coefficient (SE) | p value | Coefficient (SE) | p value | Coefficient (SE) | p value |
| Intercept | 2.44 (0.42) | <0.001 | 2.41 (0.54) | 0.002 | 2.40 (0.51) | 0.002 |
| Individual-level | Coefficient (SE) | p value | Coefficient (SE) | p value | Coefficient (SE) | p value |
| Team job crafting |  |  |  |  |  |  |
| Crafting for task | -0.08 (0.19) | 0.678 |  |  |  |  |
| Crafting for respect |  |  | -0.08 (0.11) | 0.455 |  |  |
| Crafting for information |  |  |  |  | -0.20 (0.13) | 0.125 |
| Ward-level | Coefficient (SE) | p value | Coefficient (SE) | p value | Coefficient (SE) | p value |
| Team job crafting |  |  |  |  |  |  |
| Crafting for task | -0.31 (0.57) | 0.585 |  |  |  |  |
| Crafting for respect |  |  | 0.07 (0.61) | 0.909 |  |  |
| Crafting for information |  |  |  |  | 0.16 (0.65) | 0.805 |
| Random effects | Coefficient |  | Coefficient |  | Coefficient |  |
| Intercept (Ward) | 0.12 |  | 0.10 |  | 0.09 |  |
| Residual variance | 0.87 |  | 0.89 |  | 0.90 |  |

^a^Adjusted by T1 work engagement. ^b^Adjusted by age, gender, educational status, marital status, nurse experiences, T1 work engagement, job demand, job control, supervisor support, coworker support, effort-reward imbalance. Nj = the number of wards; Ni = the number of individuals.

Table S1-2. Multilevel association between individual-level and ward-level team job crafting and work engagement among workplace social capital [low] ward at T2 (Nj= 13, Ni= 195)

|  | Model 0 (Null model) | | Model 1 (Crude model) | | Model 2 (adjusted^a^) | | Model 3 (adjusted^b^) | |
| --- | --- | --- | --- | --- | --- | --- | --- | --- |
| Fixed effects | Coefficient (SE) | p value | Coefficient (SE) | p value | Coefficient (SE) | p value | Coefficient (SE) | p value |
| Intercept | 2.42 (0.31) | <0.001 | 2.44 (0.33) | <0.001 | 2.49 (0.34) | <0.001 | 2.48 (0.34) | <0.001 |
| Individual-level | Coefficient (SE) | p value | Coefficient (SE) | p value | Coefficient (SE) | p value | Coefficient (SE) | p value |
| Team job crafting |  |  | 0.54 (0.16) | 0.001 | -0.01 (0.13) | 0.923 | 0.01 (0.14) | 0.923 |
| Crafting for task |  |  |  |  |  |  |  |  |
| Crafting for respect |  |  |  |  |  |  |  |  |
| Crafting for information |  |  |  |  |  |  |  |  |
| Ward-level | Coefficient (SE) | p value | Coefficient (SE) | p value | Coefficient (SE) | p value | Coefficient (SE) | p value |
| Team job crafting |  |  | -0.30 (0.63) | 0.636 | -0.13 (0.57) | 0.819 | -0.11 (0.57) | 0.851 |
| Crafting for task |  |  |  |  |  |  |  |  |
| Crafting for respect |  |  |  |  |  |  |  |  |
| Crafting for information |  |  |  |  |  |  |  |  |
| Random effects | Coefficient |  | Coefficient |  | Coefficient |  | Coefficient |  |
| Intercept | 0.03 |  | 0.04 |  | 0.11 |  | 0.11 |  |
| Residual variance | 0.97 |  | 0.96 |  | 0.89 |  | 0.90 |  |
| AIC | 577.687 |  | 548.720 |  | 436.263 |  | 474.254 |  |

|  | Model 3a (adjusted^b^) | | Model 3b (adjusted^b^) | | Model 3c (adjusted^b^) | |
| --- | --- | --- | --- | --- | --- | --- |
| Fixed effects | Coefficient (SE) | p value | Coefficient (SE) | p value | Coefficient (SE) | p value |
| Intercept | 2.48 (0.30) | <0.001 | 2.48 (0.35) | <0.001 | 2.51 (0.34) | <0.001 |
| Individual-level | Coefficient (SE) | p value | Coefficient (SE) | p value | Coefficient (SE) | p value |
| Team job crafting |  |  |  |  |  |  |
| Crafting for task | 0.04 (0.12) | 0.717 |  |  |  |  |
| Crafting for respect |  |  | 0.05 (0.11) | 0.630 |  |  |
| Crafting for information |  |  |  |  | -0.07 (0.12) | 0.580 |
| Ward-level | Coefficient (SE) | p value | Coefficient (SE) | p value | Coefficient (SE) | p value |
| Team job crafting |  |  |  |  |  |  |
| Crafting for task | 0.19 (0.58) | 0.739 |  |  |  |  |
| Crafting for respect |  |  | -0.13 (0.42) | 0.764 |  |  |
| Crafting for information |  |  |  |  | 0.05 (0.51) | 0.919 |
| Random effects | Coefficient |  | Coefficient |  | Coefficient |  |
| Intercept (Ward) | 0.10 |  | 0.12 |  | 0.12 |  |
| Residual variance | 0..90 |  | 0.90 |  | 0.89 |  |

^a^Adjusted by T1 work engagement. ^b^Adjusted by age, gender, educational status, marital status, nurse experiences, T1 work engagement, job demand, job control, supervisor support, coworker support, effort-reward imbalance. Nj = the number of wards; Ni = the number of individuals.

Table S1-3. Multilevel association between individual-level and ward-level team job crafting and work engagement among workplace social capital [high] ward at T3 (Nj= 17, Ni= 196)

|  | Model 0 (Null model) | | Model 1 (Crude model) | | Model 2 (adjusted^a^) | | Model 3 (adjusted^b^) | |
| --- | --- | --- | --- | --- | --- | --- | --- | --- |
| Fixed effects | Coefficient (SE) | p value | Coefficient (SE) | p value | Coefficient (SE) | p value | Coefficient (SE) | p value |
| Intercept | 2.38 (0.82) | 0.020 | 2.44 (0.86) | 0.018 | 2.39 (0.87) | 0.020 | 2.31 (0.84) | 0.032 |
| Individual-level | Coefficient (SE) | p value | Coefficient (SE) | p value | Coefficient (SE) | p value | Coefficient (SE) | p value |
| Team job crafting |  |  | 0.42 (0.25) | 0.096 | 0.05 (0.22) | 0.831 | -0.08 (0.26) | 0.751 |
|  |  |  |  |  |  |  |  |  |
|  |  |  |  |  |  |  |  |  |
|  |  |  |  |  |  |  |  |  |
| Ward-level | Coefficient (SE) | p value | Coefficient (SE) | p value | Coefficient (SE) | p value | Coefficient (SE) | p value |
| Team job crafting |  |  | -0.78 (0.87) | 0.377 | -0.51 (0.83) | 0.543 | -0.38 (0.73) | 0.601 |
|  |  |  |  |  |  |  |  |  |
|  |  |  |  |  |  |  |  |  |
|  |  |  |  |  |  |  |  |  |
| Random effects | Coefficient |  | Coefficient |  | Coefficient |  | Coefficient |  |
| Intercept | 0.14 |  | 0.16 |  | 021 |  | 0.11 |  |
| Residual variance | 0.85 |  | 0.83 |  | 0.78 |  | 0.88 |  |
| AIC | 728.836 |  | 719.576 |  | 699.870 |  | 712.193 |  |

|  | Model 3a (adjusted^b^) | | Model 3b (adjusted^b^) | | Model 3c (adjusted^b^) | |
| --- | --- | --- | --- | --- | --- | --- |
| Fixed effects | Coefficient (SE) | p value | Coefficient (SE) | p value | Coefficient (SE) | p value |
| Intercept | 2.31 (0.79) | 0.028 | 2.27 (0.90) | 0.047 | 2.29 (0.84) | 0.036 |
| Individual-level | Coefficient (SE) | p value | Coefficient (SE) | p value | Coefficient (SE) | p value |
| Team job crafting |  |  |  |  |  |  |
| Crafting for task | -0.00 (0.27) | 0.995 |  |  |  |  |
| Crafting for respect |  |  | -0.04 (0.17) | 0.810 |  |  |
| Crafting for information |  |  |  |  | -0.14 (0.22) | 0.517 |
| Ward-level | Coefficient (SE) | p value | Coefficient (SE) | p value | Coefficient (SE) | p value |
| Team job crafting |  |  |  |  |  |  |
| Crafting for task | -0.58 (0.80) | 0.472 |  |  |  |  |
| Crafting for respect |  |  | -0.09 (0.63) | 0.882 |  |  |
| Crafting for information |  |  |  |  | -0.20 (0.65) | 0.761 |
| Random effects | Coefficient |  | Coefficient |  | Coefficient |  |
| Intercept (Ward) | 0.11 |  | 0.12 |  | 0.12 |  |
| Residual variance | 0.88 |  | 0.87 |  | 0.88 |  |

^a^Adjusted by T1 work engagement. ^b^Adjusted by age, gender, educational status, marital status, nurse experiences, T1 work engagement, job demand, job control, supervisor support, coworker support, effort-reward imbalance. Nj = the number of wards; Ni = the number of individuals.

|  | Model 0 (Null model) | | Model 1 (Crude model) | | Model 2 (adjusted^a^) | | Model 3 (adjusted^b^) | |
| --- | --- | --- | --- | --- | --- | --- | --- | --- |
| Fixed effects | Coefficient (SE) | p value | Coefficient (SE) | p value | Coefficient (SE) | p value | Coefficient (SE) | p value |
| Intercept | 2.20 (0.43) | 0.001 | 2.29 (0.46) | <0.001 | 2.32 (0.46) | <0.001 | 2.32 (0.47) | <0.001 |
| Individual-level | Coefficient (SE) | p value | Coefficient (SE) | p value | Coefficient (SE) | p value | Coefficient (SE) | p value |
| Team job crafting |  |  | 0.38 (0.16) | 0.022 | -0.14 (0.17) | 0.390 | -0.13 (0.22) | 0.556 |
| Crafting for task |  |  |  |  |  |  |  |  |
| Crafting for respect |  |  |  |  |  |  |  |  |
| Crafting for information |  |  |  |  |  |  |  |  |
| Ward-level | Coefficient (SE) | p value | Coefficient (SE) | p value | Coefficient (SE) | p value | Coefficient (SE) | p value |
| Team job crafting |  |  | 0.21 (0.72) | 0.770 | 0.30 (0.65) | 0.641 | 0.21 (0.66) | 0.745 |
| Crafting for task |  |  |  |  |  |  |  |  |
| Crafting for respect |  |  |  |  |  |  |  |  |
| Crafting for information |  |  |  |  |  |  |  |  |
| Random effects | Coefficient |  | Coefficient |  | Coefficient |  | Coefficient |  |
| Intercept | 0.04 |  | 0.04 |  | 0.06 |  | 0.09 |  |
| Residual variance | 0.97 |  | 0.96 |  | 0.94 |  | 0.92 |  |
| AIC | 584.488 |  | 554.335 |  | 422.274 |  | 454.254 |  |

Table S1-4. Multilevel association between individual-level and ward-level team job crafting and work engagement among workplace social capital [low] ward at T3 (Nj= 13, Ni= 195)

|  | Model 3a (adjusted^b^) | | Model 3b (adjusted^b^) | | Model 3c (adjusted^b^) | |
| --- | --- | --- | --- | --- | --- | --- |
| Fixed effects | Coefficient (SE) | p value | Coefficient (SE) | p value | Coefficient (SE) | p value |
| Intercept | 2.27 (0.44) | <0.001 | 2.31 (0.50) | 0.001 | 2.34 (0.46) | <0.001 |
| Individual-level | Coefficient (SE) | p value | Coefficient (SE) | p value | Coefficient (SE) | p value |
| Team job crafting |  |  |  |  |  |  |
| Crafting for task | 0.03 (0.16) | 0.843 |  |  |  |  |
| Crafting for respect |  |  | -0.11 (0.16) | 0.502 |  |  |
| Crafting for information |  |  |  |  | -0.14 (0.19) | 0.449 |
| Ward-level | Coefficient (SE) | p value | Coefficient (SE) | p value | Coefficient (SE) | p value |
| Team job crafting |  |  |  |  |  |  |
| Crafting for task | -0.21 (0.68) | 0.763 |  |  |  |  |
| Crafting for respect |  |  | 0.14 (0.51) | 0.783 |  |  |
| Crafting for information |  |  |  |  | 0.39 (0.55) | 0.482 |
| Random effects | Coefficient |  | Coefficient |  | Coefficient |  |
| Intercept (Ward) | 0.08 |  | 0.09 |  | 0.08 |  |
| Residual variance | 0.92 |  | 0.92 |  | 0.93 |  |

^a^Adjusted by T1 work engagement. ^b^Adjusted by age, gender, educational status, marital status, nurse experiences, T1 work engagement, job demand, job control, supervisor support, coworker support, effort-reward imbalance. Nj = the number of wards; Ni = the number of individuals.

Table S2-1. Multilevel association between individual-level and ward-level team job crafting and work engagement among psychological safety [high] ward at T2 (Nj= 17, Ni= 180)

|  | Model 0 (Null model) | | Model 1 (Crude model) | | Model 2 (adjusted^a^) | | Model 3 (adjusted^b^) | |
| --- | --- | --- | --- | --- | --- | --- | --- | --- |
| Fixed effects | Coefficient (SE) | p value | Coefficient (SE) | p value | Coefficient (SE) | p value | Coefficient (SE) | p value |
| Intercept | 2.52 (0.41) | <0.001 | 2.58 (0.49) | <0.001 | 2.58 (0.50) | <0.001 | 2.53 (0.53) | <0.001 |
| Individual-level | Coefficient (SE) | p value | Coefficient (SE) | p value | Coefficient (SE) | p value | Coefficient (SE) | p value |
| Team job crafting |  |  | 0.37 (0.17) | 0.028 | -0.04 (0.15) | 0.778 | -0.14 (0.18) | 0.440 |
| Crafting for task |  |  |  |  |  |  |  |  |
| Crafting for respect |  |  |  |  |  |  |  |  |
| Crafting for information |  |  |  |  |  |  |  |  |
| Ward-level | Coefficient (SE) | p value | Coefficient (SE) | p value | Coefficient (SE) | p value | Coefficient (SE) | p value |
| Team job crafting |  |  | -0.67 (0.77) | 0.389 | -0.50 (0.73) | 0.493 | -0.47 (0.70) | 0.506 |
| Crafting for task |  |  |  |  |  |  |  |  |
| Crafting for respect |  |  |  |  |  |  |  |  |
| Crafting for information |  |  |  |  |  |  |  |  |
| Random effects | Coefficient |  | Coefficient |  | Coefficient |  | Coefficient |  |
| Intercept | 0.10 |  | 0.10 |  | 0.20 |  | 0.19 |  |
| Residual variance | 0.90 |  | 0.90 |  | 0.79 |  | 0.79 |  |
| AIC | 626.725 |  | 619.936 |  | 586.827 |  | 596.676 |  |

|  | Model 3a (adjusted^b^) | | Model 3b (adjusted^b^) | | Model 3c (adjusted^b^) | |
| --- | --- | --- | --- | --- | --- | --- |
| Fixed effects | Coefficient (SE) | p value | Coefficient (SE) | p value | Coefficient (SE) | p value |
| Intercept | 2.54 (0.44) | <0.001 | 2.47 (0.54) | <0.001 | 2.50 (0.53) | <0.001 |
| Individual-level | Coefficient (SE) | p value | Coefficient (SE) | p value | Coefficient (SE) | p value |
| Team job crafting |  |  |  |  |  |  |
| Crafting for task | -0.10 (0.16) | 0.566 |  |  |  |  |
| Crafting for respect |  |  | -0.04 (0.13) | 0.735 |  |  |
| Crafting for information |  |  |  |  | -0.17 (0.18) | 0.348 |
| Ward-level | Coefficient (SE) | p value | Coefficient (SE) | p value | Coefficient (SE) | p value |
| Team job crafting |  |  |  |  |  |  |
| Crafting for task | -0.70 (0.61) | 0.258 |  |  |  |  |
| Crafting for respect |  |  | -0.15 (0.59) | 0.805 |  |  |
| Crafting for information |  |  |  |  | -0.21 (0.67) | 0.752 |
| Random effects | Coefficient |  | Coefficient |  | Coefficient |  |
| Intercept (Ward) | 0.17 |  | 0.19 |  | 0.21 |  |
| Residual variance | 0.83 |  | 0.79 |  | 0.77 |  |

^a^Adjusted by T1 work engagement. ^b^Adjusted by age, gender, educational status, marital status, nurse experiences, T1 work engagement, job demand, job control, supervisor support, coworker support, effort-reward imbalance. Nj = the number of wards; Ni = the number of individuals.

Table S2-2. Multilevel association between individual-level and ward-level team job crafting and work engagement among psychological safety [low] ward at T2 (Nj= 13, Ni= 211)

|  | Model 0 (Null model) | | Model 1 (Crude model) | | Model 2 (adjusted^a^) | | Model 3 (adjusted^b^) | |
| --- | --- | --- | --- | --- | --- | --- | --- | --- |
| Fixed effects | Coefficient (SE) | p value | Coefficient (SE) | p value | Coefficient (SE) | p value | Coefficient (SE) | p value |
| Intercept | 2.46 (0.29) | <0.001 | 2.48 (0.29) | <0.001 | 2.49 (0.29) | <0.001 | 2.49 (0.29) | <0.001 |
| Individual-level | Coefficient (SE) | p value | Coefficient (SE) | p value | Coefficient (SE) | p value | Coefficient (SE) | p value |
| Team job crafting |  |  | 0.50 (0.15) | 0.002 | -0.06 (0.13) | 0.652 | -0.07 (0.14) | 0.600 |
| Crafting for task |  |  |  |  |  |  |  |  |
| Crafting for respect |  |  |  |  |  |  |  |  |
| Crafting for information |  |  |  |  |  |  |  |  |
| Ward-level | Coefficient (SE) | p value | Coefficient (SE) | p value | Coefficient (SE) | p value | Coefficient (SE) | p value |
| Team job crafting |  |  | -0.22 (0.54) | 0.685 | -0.09 (0.44) | 0.847 | -0.08 (0.45) | 0.855 |
| Crafting for task |  |  |  |  |  |  |  |  |
| Crafting for respect |  |  |  |  |  |  |  |  |
| Crafting for information |  |  |  |  |  |  |  |  |
| Random effects | Coefficient |  | Coefficient |  | Coefficient |  | Coefficient |  |
| Intercept | 0.03 |  | 0.03 |  | 0.07 |  | 0.07 |  |
| Residual variance | 0.97 |  | 0.96 |  | 0.95 |  | 0.95 |  |
| AIC | 724.344 |  | 700.757 |  | 676.950 |  | 709.720 |  |

|  | Model 3a (adjusted^b^) | | Model 3b (adjusted^b^) | | Model 3c (adjusted^b^) | |
| --- | --- | --- | --- | --- | --- | --- |
| Fixed effects | Coefficient (SE) | p value | Coefficient (SE) | p value | Coefficient (SE) | p value |
| Intercept | 2.50 (0.29) | <0.001 | 2.50 (0.29) | <0.001 | 2.51 (0.28) | <0.001 |
| Individual-level | Coefficient (SE) | p value | Coefficient (SE) | p value | Coefficient (SE) | p value |
| Team job crafting |  |  |  |  |  |  |
| Crafting for task | 0.03 (0.12) | 0.821 |  |  |  |  |
| Crafting for respect |  |  | -0.04 (0.11) | 0.721 |  |  |
| Crafting for information |  |  |  |  | -0.13 (0.12) | 0.265 |
| Ward-level | Coefficient (SE) | p value | Coefficient (SE) | p value | Coefficient (SE) | p value |
| Team job crafting |  |  |  |  |  |  |
| Crafting for task | -0.12 (0.46) | 0.790 |  |  |  |  |
| Crafting for respect |  |  | -0.06 (0.32) | 0.845 |  |  |
| Crafting for information |  |  |  |  | 0.02 (0.39) | 0.954 |
| Random effects | Coefficient |  | Coefficient |  | Coefficient |  |
| Intercept (Ward) | 0.07 |  | 0.07 |  | 0.07 |  |
| Residual variance | 0.95 |  | 0.95 |  | 0.95 |  |

^a^Adjusted by T1 work engagement. ^b^Adjusted by age, gender, educational status, marital status, nurse experiences, T1 work engagement, job demand, job control, supervisor support, coworker support, effort-reward imbalance. Nj = the number of wards; Ni = the number of individuals.

Table S2-3. Multilevel association between individual-level and ward-level team job crafting and work engagement among psychological safety [high] ward at T3 (Nj= 17, Ni= 180)

|  | Model 0 (Null model) | | Model 1 (Crude model) | | Model 2 (adjusted^a^) | | Model 3 (adjusted^b^) | |
| --- | --- | --- | --- | --- | --- | --- | --- | --- |
| Fixed effects | Coefficient (SE) | p value | Coefficient (SE) | p value | Coefficient (SE) | p value | Coefficient (SE) | p value |
| Intercept | 2.40 (0.79) | 0.020 | 2.48 (0.81) | 0.011 | 2.39 (0.77) | 0.016 | 2.39 (0.77) | 0.015 |
| Individual-level | Coefficient (SE) | p value | Coefficient (SE) | p value | Coefficient (SE) | p value | Coefficient (SE) | p value |
| Team job crafting |  |  | 0.41 (0.25) | 0.103 | -0.10 (0.26) | 0.692 | -0.10 (0.26) | 0.692 |
| Crafting for task |  |  |  |  |  |  |  |  |
| Crafting for respect |  |  |  |  |  |  |  |  |
| Crafting for information |  |  |  |  |  |  |  |  |
| Ward-level | Coefficient (SE) | p value | Coefficient (SE) | p value | Coefficient (SE) | p value | Coefficient (SE) | p value |
| Team job crafting |  |  | -0.85 (0.81) | 0.293 | -0.45 (0.71) | 0.527 | -0.45 (0.71) | 0.527 |
| Crafting for task |  |  |  |  |  |  |  |  |
| Crafting for respect |  |  |  |  |  |  |  |  |
| Crafting for information |  |  |  |  |  |  |  |  |
| Random effects | Coefficient |  | Coefficient |  | Coefficient |  | Coefficient |  |
| Intercept | 0.15 |  | 0.17 |  | 0.15 |  | 0.07 |  |
| Residual variance | 0.85 |  | 0.82 |  | 0.84 |  | 0.92 |  |
| AIC | 684.136 |  | 672.878 |  | 651.980 |  | 665.273 |  |

|  | Model 3a (adjusted^b^) | | Model 3b (adjusted^b^) | | Model 3c (adjusted^b^) | |
| --- | --- | --- | --- | --- | --- | --- |
| Fixed effects | Coefficient (SE) | p value | Coefficient (SE) | p value | Coefficient (SE) | p value |
| Intercept | 2.40 (0.72) | 0.013 | 2.33 (0.82) | 0.029 | 2.36 (0.76) | 0.015 |
| Individual-level | Coefficient (SE) | p value | Coefficient (SE) | p value | Coefficient (SE) | p value |
| Team job crafting |  |  |  |  |  |  |
| Crafting for task | -0.03 (0.33) | 0.923 |  |  |  |  |
| Crafting for respect |  |  | -0.05 (0.18) | 0.760 |  |  |
| Crafting for information |  |  |  |  | -0.15 (0.21) | 0.460 |
| Ward-level | Coefficient (SE) | p value | Coefficient (SE) | p value | Coefficient (SE) | p value |
| Team job crafting |  |  |  |  |  |  |
| Crafting for task | -0.68 (0.85) | 0.424 |  |  |  |  |
| Crafting for respect |  |  | -0.11 (0.53) | 0.839 |  |  |
| Crafting for information |  |  |  |  | -0.27 (0.66) | 0.684 |
| Random effects | Coefficient |  | Coefficient |  | Coefficient |  |
| Intercept (Ward) | 0.07 |  | 0.07 |  | 0.08 |  |
| Residual variance | 0.93 |  | 0.92 |  | 0.91 |  |

^a^Adjusted by T1 work engagement. ^b^Adjusted by age, gender, educational status, marital status, nurse experiences, T1 work engagement, job demand, job control, supervisor support, coworker support, effort-reward imbalance. Nj = the number of wards; Ni = the number of individuals.

Table S2-4. Multilevel association between individual-level and ward-level team job crafting and work engagement among psychological safety [low] ward at T3 (Nj= 13, Ni= 211)

|  | Model 0 (Null model) | | Model 1 (Crude model) | | Model 2 (adjusted^a^) | | Model 3 (adjusted^b^) | |
| --- | --- | --- | --- | --- | --- | --- | --- | --- |
| Fixed effects | Coefficient (SE) | p value | Coefficient (SE) | p value | Coefficient (SE) | p value | Coefficient (SE) | p value |
| Intercept | 2.20 (0.37) | <0.001 | 2.27 (0.38) | <0.001 | 2.27 (0.38) | <0.001 | 2.33 (0.38) | <0.001 |
| Individual-level | Coefficient (SE) | p value | Coefficient (SE) | p value | Coefficient (SE) | p value | Coefficient (SE) | p value |
| Team job crafting |  |  | 0.46 (0.15) | 0.003 | -0.09 (0.14) | 0.512 | -0.11 (0.16) | 0.508 |
| Crafting for task |  |  |  |  |  |  |  |  |
| Crafting for respect |  |  |  |  |  |  |  |  |
| Crafting for information |  |  |  |  |  |  |  |  |
| Ward-level | Coefficient (SE) | p value | Coefficient (SE) | p value | Coefficient (SE) | p value | Coefficient (SE) | p value |
| Team job crafting |  |  | 9.03 (0.59) | 0.958 | 0.14 (0.50) | 0.787 | 0.10 (0.49) | 0.843 |
| Crafting for task |  |  |  |  |  |  |  |  |
| Crafting for respect |  |  |  |  |  |  |  |  |
| Crafting for information |  |  |  |  |  |  |  |  |
| Random effects | Coefficient |  | Coefficient |  | Coefficient |  | Coefficient |  |
| Intercept | 0.05 |  | 0.06 |  | 0.08 |  | 0.07 |  |
| Residual variance | 0.95 |  | 0.93 |  | 0.92 |  | 0.93 |  |
| AIC | 578.517 |  | 552.785 |  | 445.659 |  | 475.003 |  |

|  | Model 3a (adjusted^b^) | | Model 3b (adjusted^b^) | | Model 3c (adjusted^b^) | |
| --- | --- | --- | --- | --- | --- | --- |
| Fixed effects | Coefficient (SE) | p value | Coefficient (SE) | p value | Coefficient (SE) | p value |
| Intercept | 2.29 (0.38) | <0.001 | 2.33 (0.38) | <0.001 | 2.35 (0.36) | <0.001 |
| Individual-level | Coefficient (SE) | p value | Coefficient (SE) | p value | Coefficient (SE) | p value |
| Team job crafting |  |  |  |  |  |  |
| Crafting for task | 0.11 (0.12) | 0.990 |  |  |  |  |
| Crafting for respect |  |  | -0.10 (0.12) | 0.447 |  |  |
| Crafting for information |  |  |  |  | -0.13 (0.14) | 0.353 |
| Ward-level | Coefficient (SE) | p value | Coefficient (SE) | p value | Coefficient (SE) | p value |
| Team job crafting |  |  |  |  |  |  |
| Crafting for task | -0.29 (0.56) | 0.603 |  |  |  |  |
| Crafting for respect |  |  | 0.10 (0.36) | 0.791 |  |  |
| Crafting for information |  |  |  |  | 0.27 (0.40) | 0.507 |
| Random effects | Coefficient |  | Coefficient |  | Coefficient |  |
| Intercept (Ward) | 0.07 |  | 0.07 |  | 0.07 |  |
| Residual variance | 0.94 |  | 0.93 |  | 0.93 |  |

^a^Adjusted by T1 work engagement. ^b^Adjusted by age, gender, educational status, marital status, nurse experiences, T1 work engagement, job demand, job control, supervisor support, coworker support, effort-reward imbalance. Nj = the number of wards; Ni = the number of individuals.
